# The RGD motif is involved in CD97/ADGRE5-promoted cell adhesion and viability of HT1080 cells

**DOI:** 10.1038/s41598-018-38045-w

**Published:** 2019-02-06

**Authors:** Wen-Ye Tjong, Hsi-Hsien Lin

**Affiliations:** 1 Graduate Institute of Biomedical Sciences, College of Medicine, Chang Gung University, Taoyuan, Taiwan; 2 Department of Microbiology and Immunology, College of Medicine, Chang Gung University, Taoyuan, Taiwan; 3 Department of Anatomic Pathology, Chang Gung Memorial Hospital-Linkou, Taoyuan, Taiwan; 4 Chang Gung Immunology Consortium, Chang Gung Memorial Hospital-Linkou, Taoyuan, Taiwan

## Abstract

CD97/ADGRE5 is an adhesion G protein-coupled receptor (aGPCR) involved in tumor cell adhesion, migration, angiogenesis, and apoptosis. CD97 has been shown previously to stimulate angiogenesis by interacting with integrins on endothelial cells via an Arginine-Glycine-Aspartic acid (RGD) motif. In this report, the role of the RGD motif in tumor cell adhesion and apoptosis was investigated using a previously-established HT1080 cell-based system. We found that the RGD motif is critical in CD97-promoted cell adhesion, in part due to the up-regulation of αvβ5 and α2β1 integrins, and that CD97 mediates its anti-apoptotic effect in extrinsic apoptosis via RGD-dependent cell adhesion. In contrast, CD97-modulated anti-apoptotic effect in intrinsic apoptosis is mediated by RGD-independent, N-cadherin-induced homotypic cell aggregation. Hence, CD97 promotes tumorigenesis via RGD-dependent and -independent mechanisms.

## Introduction

Adhesion-class G protein-coupled receptors (aGPCRs) are evolutionarily conserved cell surface proteins characterized by a long N-terminal extracellular domain (ECD) linked to a seven-span transmembrane (7TM) region. The ECD of aGPCRs is involved in cellular adhesion and normally contains distinct adhesion protein motifs, such as epidermal growth factor (EGF)-like, thrombospondin-like and cadherin-like domains^1^. A conserved GPCR-Autoproteolysis INducing (GAIN) domain usually follows immediately after the cell-adhesion domains and most aGPCRs are dissected at the GPCR proteolytic site (GPS) of the GAIN domain by a self-catalytic post-translational proteolytic cleavage event^2,3^. However, the two cleaved receptor fragments usually do not separate but remain as a non-covalent complex. Therefore, a mature aGPCR typically consists of an extracellular subunit (N-terminal fragment, NTF) associated with a 7TM subunit (C-terminal fragment, CTF)^1^.

GPCRs, including aGPCRs, have been linked to cancer progression^4–9^. CD97/ADGRE5 is a member of the ADGRE (EGF-TM7) family of aGPCRs, which are characterized by multiple EGF-like repeats in the ECD^10^. Due to alternative splicing, three CD97 receptor isoforms containing different EGF-like motifs, namely CD97(EGF/125), CD97(EGF/1235) and CD97(EGF/1–5), were identified^11^. These distinct CD97 isoforms interact with four endogenous cellular ligands including CD55 (DAF), α5β1 integrin, CD90 (Thy-1), and chondroitin sulphate mostly in an isoform-restricted manner^12–15^. Nevertheless, the integrin α5β1 is thought to interact with all CD97 isoforms through the Arginine-Glycine-Aspartic acid (RGD) motif located in the GAIN domain^15^.

CD97 was identified originally as an early activation marker of lymphocytes^16^, but later found also abundantly on granulocytes, monocytes/macrophages and smooth muscle cells^11,17,18^. In addition, CD97 is highly expressed in various cancerous tissues including esophageal, colorectal, gastric, thyroid, and pancreatic carcinomas (reviewed in^19^). Importantly, higher CD97 expression levels are usually detected in the disseminated/scattered cells at the tumor invasion fronts and patients with more CD97-positive scattered tumor cells tend to have a poorer prognosis and enhanced lymph vessel invasion^20^. Previous studies by us and others have shown a functional link of CD97 to tumor cell adhesion, motility, metastasis, angiogenesis, and apoptosis^15,21–23^.

Of note, studies have shown that the CD97-NTF is able to promote angiogenesis in part by binding to the α5β1 and αvβ3 integrins on human umbilical vein endothelial cells (HUVECs) via its RGD motif^15^. Interestingly, the RGD motif was not present in rodent CD97 molecules. In fact, only certain primates such as human, gorilla, and chimpanzee, but not monkey and orangutan CD97 receptors contain the RGD sequence. This suggests a possible unique function for the RGD motif in the primate CD97 receptors.

As the RGD peptide itself is a well-known cell-adhesion motif capable of modulating numerous cellular functions^24,25^, we herein aim to delineate the possible role of the RGD motif in CD97-modulated tumor cell adhesion and apoptosis. To this end, we adapted the previously-established HT1080 cell-based experimental system utilizing site-directed mutants, chimeric receptors, and specific function-blocking peptides. Our results reveal a critical role for the RGD motif in CD97-promoted tumor cell adhesion. The anti-apoptotic effect of CD97 is mediated via RGD-dependent and RGD-independent processes in the extrinsic and intrinsic apoptotic pathways, respectively. These findings contribute to the functional insights of CD97-regulated tumorigenesis.

## Results

### Generation of stable HT1080 cell lines expressing recombinant CD97 and EGF-like module-containing mucin-like hormone receptor-like 2 (EMR2) receptors

**Figure 1:**
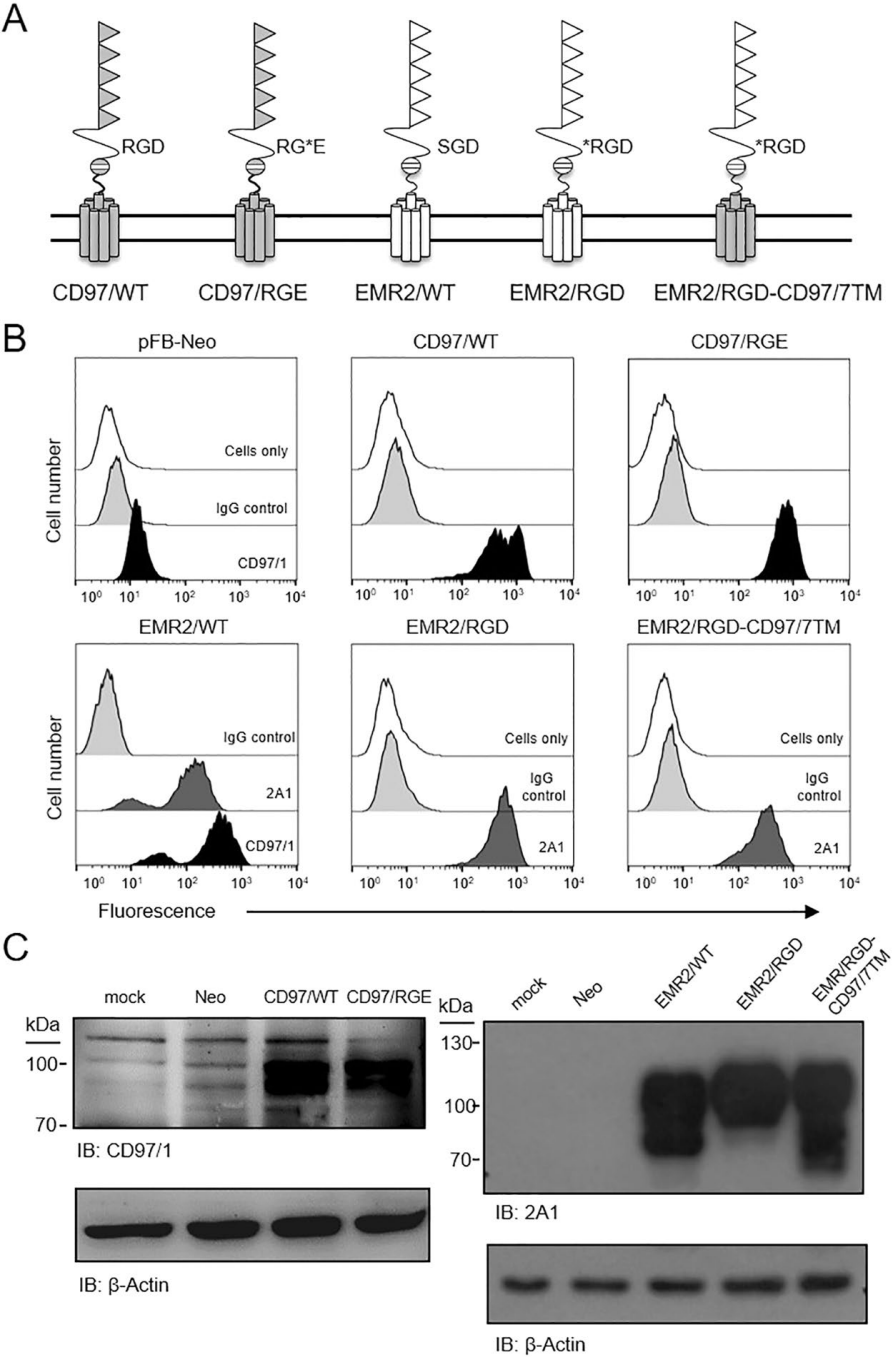
Generation of HT1080 cells stably expressing genetically engineered CD97 and EMR2 proteins (**A**) Schematic representation of the CD97 (grey background) and EMR2 (white background) receptors studied in this study. The triangles represent EGF-like domains, while the broken circle and the cylinders indicate the GPS and the 7TM regions, respectively. Upper case letters indicate the specific amino acids of interest in the corresponding proteins. *Represents the single residue mutated in the receptor. (**B**,**C**) The expression of CD97 and EMR2 molecules in HT1080 stably transfected cells was confirmed by flow cytometry (**B**) and western blotting (**C**) analyses using appropriate mAbs as indicated. Cells expressing distinct CD97 and EMR2 proteins were numbered individually as shown in (**C**). pFB-Neo indicates control stable HT1080 cells transduced by the empty pFB-Neo vector.

In accordance with our previous experimental model^22,23^, stable HT1080 cell lines expressing CD97/WT, CD97/RGE, EMR2/WT, EMR2/RGD, and EMR2/RGD-CD97/7TM proteins were established to examine the role of the RGD motif in the tumorigenic functions mediated by CD97. EMR2 is included as a control because it shares with CD97 a 97% sequence identity in the EGF-like domains, but does not contain a RGD motif in its GAIN domain^26^. To this end, the CD97 and EMR2 receptor isoforms containing the full-length 5 EGF motifs were investigated. In addition to the wild-type (WT) proteins, we mutated the RGD sequence in the CD97 GAIN domain to RGE (CD97/RGE) and similarly changed a corresponding SGD sequence in the EMR2 GAIN domain to RGD (EMR2/RGD). Finally, EMR2/RGD-CD97/7TM is a chimeric receptor containing the extracellular EMR2/RGD linked to the CD97/7TM region (Fig. 1A). Flow cytometry and western blot analyses verified the expression of the recombinant CD97 and EMR2 proteins was up-regulated in the designated groups of stable HT1080 cells to a consistent and comparable level when compared to the control HT1080-Neo cells (Fig. 1B,C).

### The RGD motif of CD97 is involved in promoting cell adhesion by up-regulating the expression of integrin αvβ5 and α2β1

**Figure 2:**
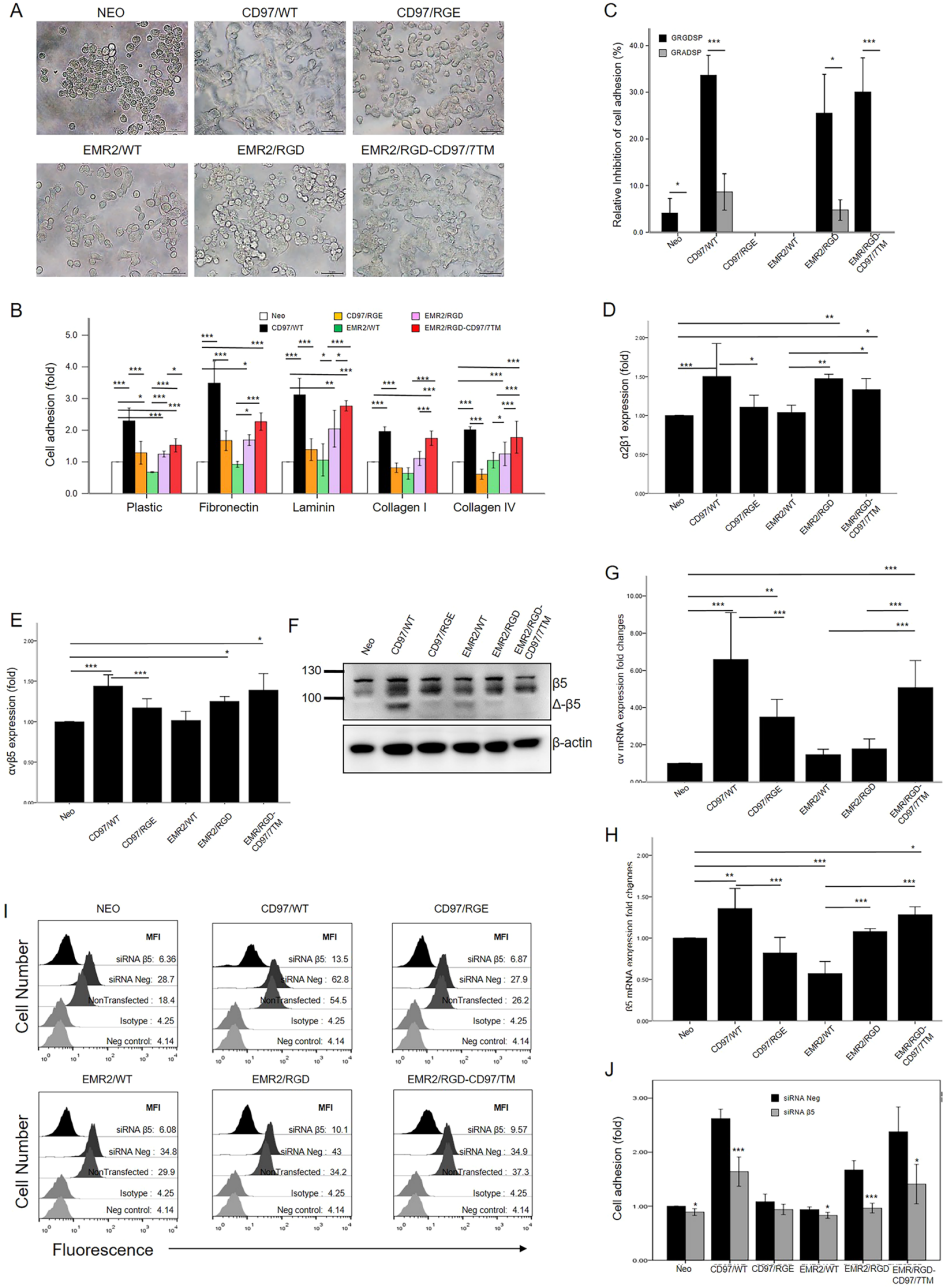
Enhanced HT1080 cell adhesion induced by CD97 and EMR2 is dependent in part on the RGD motif. (**A**) Microscopy observation of HT1080 stable cells adhered to culture plastics. The number represents distinct groups of stable cells as indicated in (**B**). Scale bar: 50 μm. (**B**) Quantification of cell adhesion of variable HT1080 stable cells to plates coated without or with the ECM proteins as indicated (N = 3, mean ± SD; *P < 0.05, **P < 0.01, and ***P < 0.005). (**C**) Mean percentage inhibition of cell adhesion by the GRGDSP peptide (50 µg/ml). The GRADSP peptide (50 µg/ml) acts as a negative control (N = 4, mean ± SD; *P < 0.05, and ***P < 0.005, determined by two-tailed student’s t-test). The relative expression level of the integrin α2β1 (**D**) and αvβ5 (**E**) on different HT1080 stable cells was analyzed by flow cytometry (N = 3, mean ± SD; *P < 0.05, and ***P < 0.005). (**F**) The total cell lysates were analysed by western blotting using mAb αvβ5. (**G**, **H**) The level of mRNA expression of αv and β5 were analyzed by RT-qPCR (N = 3, mean ± SD; *P < 0.05, and ***P < 0.005. (**I**) FACS analysis was used to check the knockdown efficiency of siRNA β5. This figure is the representative of three independent experiments. (**J**) The adhesion assay on culture plastic was performed after knockdown of β5 integrin (N = 4, mean ± SD; *P < 0.05, and ***P < 0.005, determined by two-tailed Student’s t-test). (The exact p-value numbers for each comparison are listed in the supplemental materials).

We have shown previously that CD97 expression in HT1080 cells promotes cell adhesion by the up-regulation of integrins^23^. To investigate the potential involvement of the RGD tri-peptide in CD97-promoted cell adherence, the established stable HT1080 cells were subjected to the cell adhesion assay on tissue culture plates coated without or with various extracellular matrix (ECM) proteins (Fig. 2). As shown in Fig. 2A, serum-starved cells expressing CD97/WT, EMR2/RGD, and EMR2/RGD-CD97/7TM displayed enhanced adhesion and more elongated and spreading morphology on plastic plates whereas the control HT1080-Neo cells and other stable cells tended to form compact cell clusters with a less adhesive phenotype. Importantly, the enhanced adhesion and spreading of CD97/WT, EMR2/RGD, and EMR2/RGD-CD97/7TM stable cells was also observed on plates coated with fibronectin, laminin, collagen I, and collagen IV (Fig. 2B). Interestingly, the increased adhesion was most prominent in cells expressing CD97/WT and EMR2/RGD-CD97/7TM on all surfaces, while the EMR2/RGD stable cells seemed to prefer certain substratum such as fibronectin and laminin. These data are consistent with our previous results and suggest a critical role of the RGD motif in CD97-promoted HT1080 cell adhesion^23^. Furthermore, it also reveals a gain-of-function for EMR2 in promoting cell adhesion by simply changing one residue from the SGD to RGD.

Next, the GRGDSP peptide was used to block the RGD motif to further verify its involvement in promoting cell adhesion. As shown, the GRGDSP peptide blocked the adhesion efficiency of cells expressing CD97/WT (~34%), EMR2/RGD (~25%), and EMR2 RGD-CD97/7TM (~30%), but has no apparent effect on the adhesion of control and other stable cells (Fig. 2C). By contrast, the negative control GRADSP peptide showed no any inhibitory effect on cell adhesion on all cells tested. The RGD peptide is known to inhibit cell adhesion by competing with integrin-matrix interaction, while integrins are known to display different conformations and ligand-binding affinities. We hence evaluated the expression levels and the activation status of multiple integrin molecules including α2β1, αvβ3, αvβ5, β1, and α3 on the surface of stable HT1080 cells by FACS, western blotting, and qRT-PCR analyses (Fig. 2D–H, Supplementary Figs 1 and 2). Among the selective integrin proteins examined, the α2β1 and αvβ5 integrins were found to be up-regulated consistently and significantly in cells expressing CD97/WT, EMR2/RGD, and EMR2/RGD-CD97/7TM (Fig. 2D–H). Furthermore, siRNA-mediated specific knock down of the β5 integrin significantly attenuated the enhanced cell adhesion (Fig. 2I,J). As the α2β1 and αvβ5 integrins are well known to interact with multiple ECM proteins such as FN, Col-I, and -IV^27–32^, this finding indicates that the enhanced cell adhesion observed in the selected stable cells might be mediated in part by the up-regulated α2β1and αvβ5 integrins. Importantly, the up-regulation of these two integrins seems to be dependent on the presence of the RGD motif on CD97 and EMR2.

### The RGD motif of CD97 is involved in the inhibition of cell apoptosis

**Figure 3:**
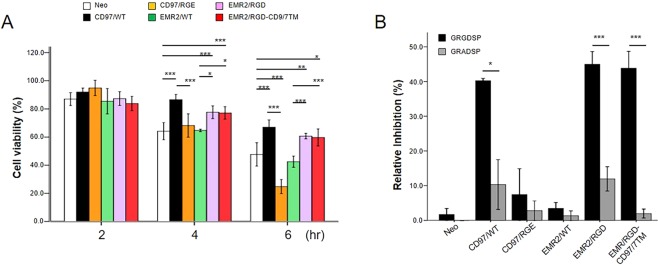
The RGD motif of CD97 promotes cell viability. (**A**) Cell viability was determined at different time points (2, 4, and 6 h) following the induction of cell apoptosis by incubation in the presence of cycloheximide (10 µg/ml) and TNF-α (5 ng/ml). (**B**) The effect of exogenously added GRGDSP and GRADSP peptide (50 µg/ml each) in inhibiting cell survival under apoptotic condition induced by the cycloheximide and TNF-α treatment for 4 h. (N = 3, mean ± SD; *P < 0.05, and ***P < 0.005). (The exact p-value numbers for each comparison are listed in the supplemental materials).

CD97 has recently been shown to inhibit cell apoptosis^22^. Interestingly, the exogenous RGD peptide is known to promote apoptosis of human endothelial cells through the activation of caspase 8 and caspase 9^33^. These results prompted us to investigate the possible involvement of the RGD motif in CD97-inhibited apoptosis. Cell apoptosis was induced by incubating cells in the presence of TNF-α and cyclocheximide (TNF/CHX) that induces the extrinsic apoptotic pathway. Cell viability assay indicated that all stable cells were viable at 2 h incubation, however approximately 35% of HT1080-Neo control cells and cells expressing CD97/RGE and EMR2/WT were dead at 4 h incubation. When analysed at 6 h incubation, more than 55% of the control cells, CD97/RGE- and EMR2/WT-expressing cells were dead, while ~40% of CD97/WT-, EMR2/RGD-, and EMR2/RGD-CD97/7TM expressing cells were dead (Fig. 3A). These results suggest that the RGD motif on CD97 and EMR2 is involved in promoting viability of HT1080 cells undergoing extrinsic apoptosis. To verify this, the GRGDSP peptide was added in the presence of TNF/CHX. Cell viability assay showed that the GRGDSP peptide, but not the control GRADSP peptide, inhibits significantly the viability of cells expressing CD97/WT (~40%), EMR2/RGD (~45%), and EMR2/RGD-CD97/7TM (~44%). By contrast, both peptides have no apparent effect on the viability of the control cells and those expressing CD97/RGE and EMR2/WT (Fig. 3B).

**Figure 4:**
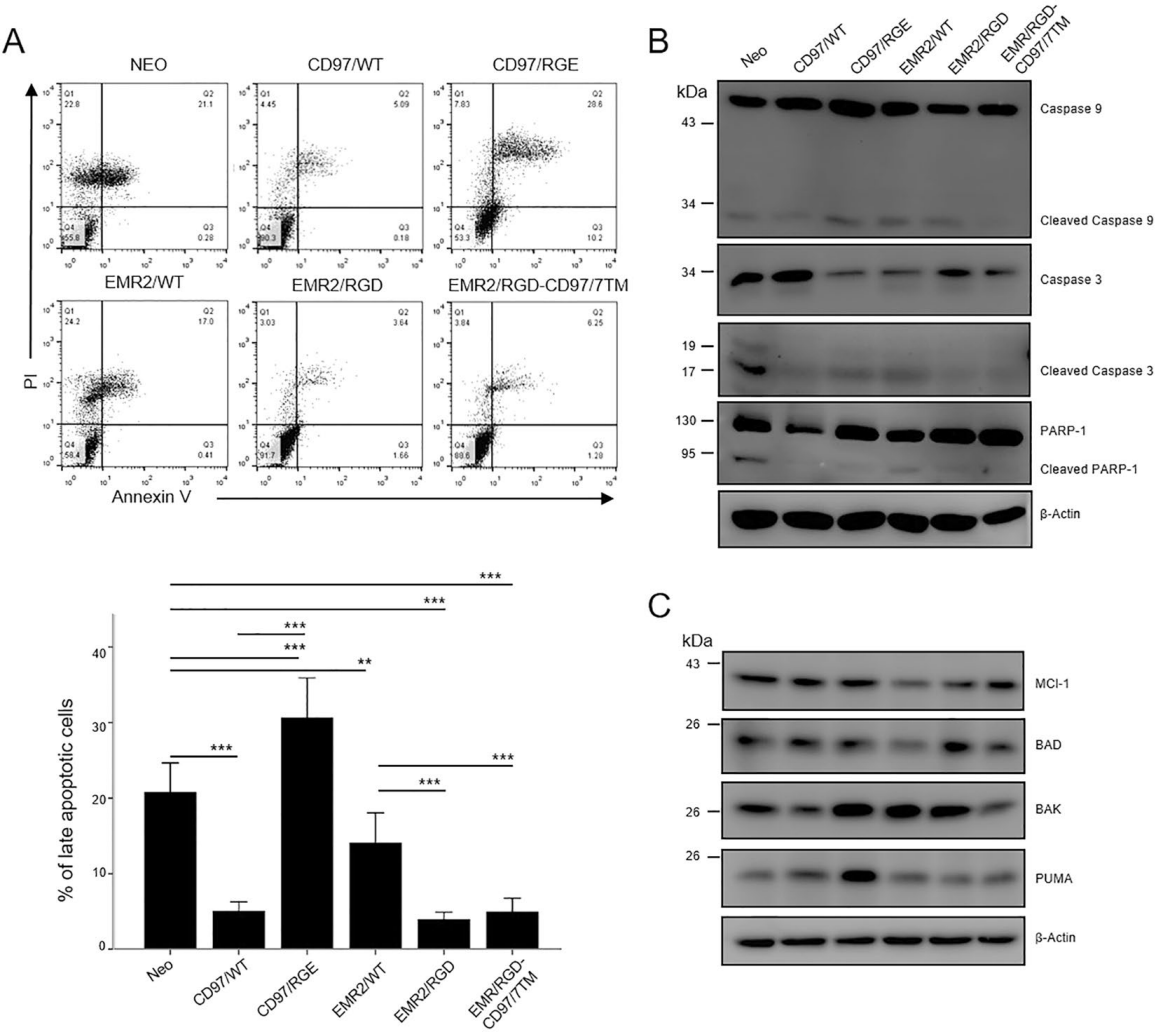
The RGD motif of CD97 inhibits extrinsically-induced apoptosis. (**A**) The flow cytometry analysis of cell apoptosis of indicated stable HT1080 cells cultured in the presence of cycloheximide (10 µg/ml) and TNF-α (5 ng/ml) for 6 h. Top panel: the staining patterns of Annexin V (AV) and Propidium Iodide (PI) as determined by the FACS analysis. Bottom panel: a graph showing the percentage of late apoptotic (AV^+^/PI^+^) cells (N = 4, mean ± SD; *P < 0.05 and **P < 0.01). (**B**,**C**) Western blotting analysis of various pro- and anti-apoptotic molecules in HT1080 stable cells cultured in the presence of cycloheximide (10 µg/ml) and TNF-α (5 ng/ml) for 6 h. β-actin was a loading control. (The exact p-value numbers for each comparison are listed in the supplemental materials).

**Figure 5:**
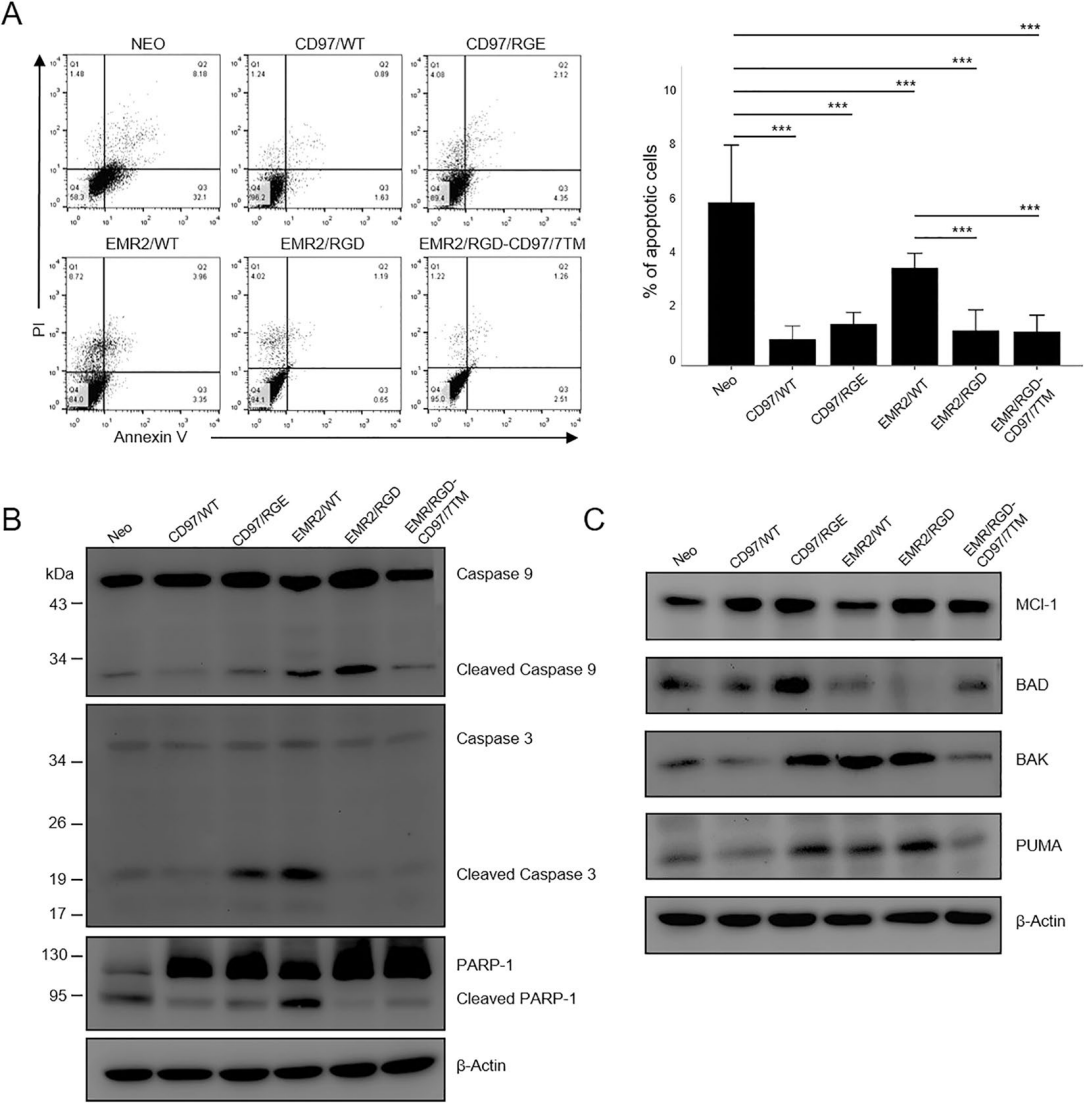
The RGD motif of EMR2 inhibits intrinsically-induced apoptosis. (**A**) Flow cytometry analysis of cell apoptosis of indicated stable HT1080 cells cultured in serum-free medium for 48 h. Left panel: the staining patterns of Annexin V (AV) and Propidium Iodide (PI) as determined by the FACS analysis. Right panel: a graph showing the percentage of late apoptotic (AV^+^/PI^+^) cells (N = 4, mean ± SD; *P < 0.05 and ***P < 0.005). (**B**,**C**) Western blotting analysis of various pro- and anti-apoptotic molecules in HT1080 stable cells cultured in serum-free medium for 48 h. β-actin was a loading control. (The exact p-value numbers for each comparison are listed in the supplemental materials).

To investigate further the anti-apoptotic role of the RGD motif, another type of apoptosis, the intrinsic apoptotic pathway, was induced by culturing cells in serum-deprived condition for 48 h. Cell apoptosis induction by both the extrinsic and intrinsic pathways were confirmed by the flow cytometry analysis of Annexin V (AV) and propidium iodide (PI) staining, as well as by the western blot analysis of protein markers of cell apoptosis including caspase 9, caspase 3, PARP-1, MCl-1, BAD, BAK, and PUMA. Consistently, the FACS analysis showed that the HT1080-Neo control cells and cells expressing EMR2/WT displayed a higher percentage of apoptotic cell populations than cells expressing CD97/WT, EMR2/RGD, and EMR2/RGD-CD97/7TM in both extrinsic and intrinsic apoptotic conditions (Figs 4A and 5A). Likewise, the western blot analysis showed that the CD97/WT, EMR2/RGD, and EMR2 RGD-CD97/7TM expressing cells expressed lower levels of cleaved caspase-9, caspase-3, and PARP-1 proteins (Figs 4B and 5B). In addition, the expression of the anti-apoptotic protein MCl-1 was significantly up-regulated in HT1080 cells expressing CD97/WT, EMR2/RGD, and EMR2/RGD-CD97/7TM when compared to HT1080-Neo cells and EMR2/WT-expressing cells (Figs 4C and 5C). On the other hand, the expression of pro-apoptotic proteins (BAD, BAK, and PUMA) was quite variable among the different groups of stable cells (Figs 4C and 5C). Of note, the FACS and western blot analyses of CD97/RGE-expressing cells showed somewhat differential results in both apoptotic conditions. Hence, in comparison to cells expressing CD97/WT, CD97/RGE-expressing cells were less viable under extrinsic apoptotic conditions, but displayed a similar survival advantage in intrinsic apoptotic conditions (Figs 4 and 5). Taken together, these results indicate that the RGD motif in CD97 and EMR2 is critical in the anti-apoptotic function of HT1080 cells through the selective modulation of apoptosis-related proteins.

### The RGD motif of EMR2 is involved in N-cadherin up-regulation and cell aggregation

**Figure 6:**
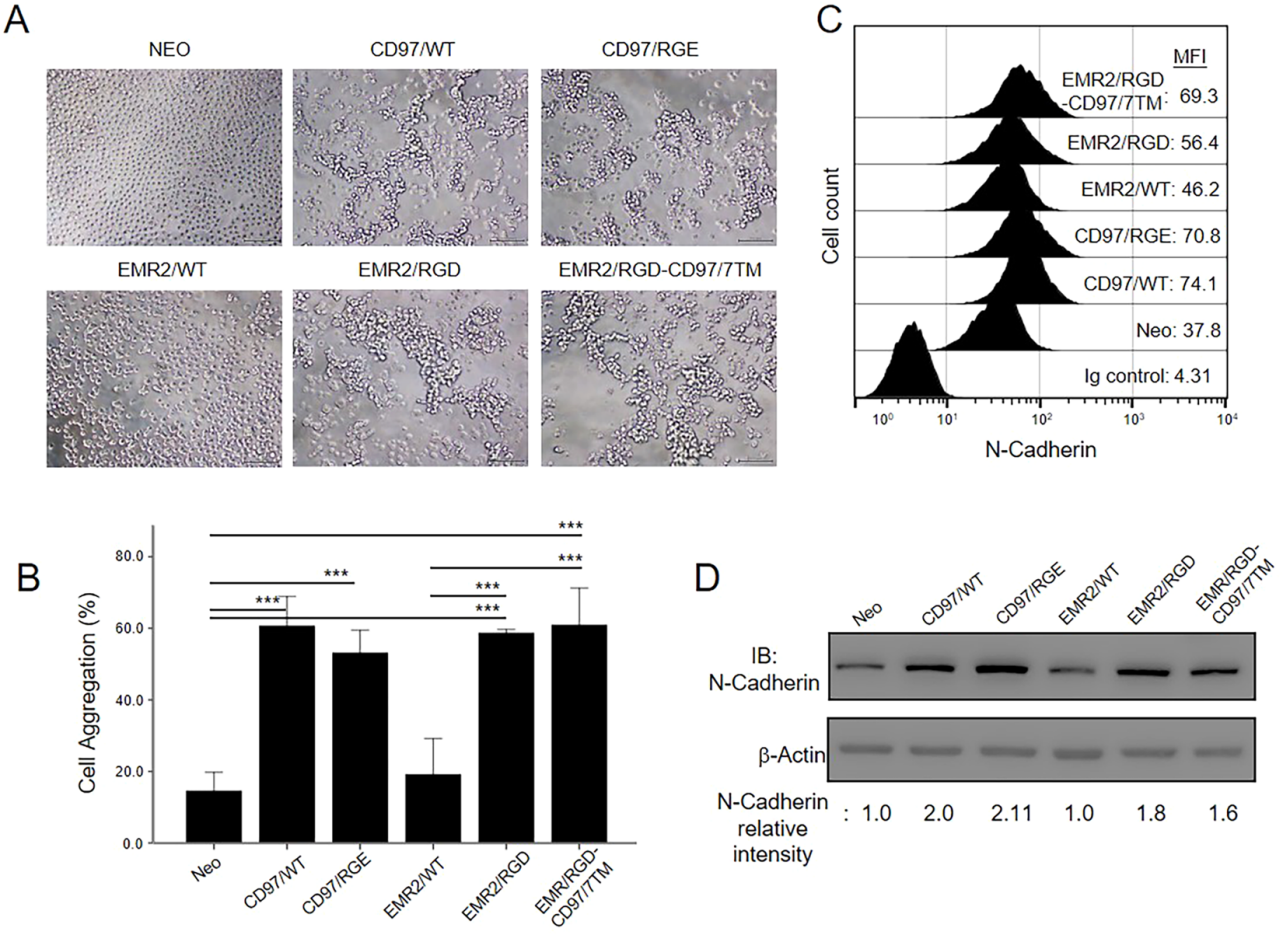
The RGD motif of EMR2 promotes N-cadherin expression resulting in enhanced HT1080 cell aggregation. Serum-starved HT1080 stable cells were cultured in serum-free conditions favoring cell aggregation for 1 h. (A) Microscopy observation of homotypic cell aggregation. Scale bar: 100 μm. (**B**) Graph showing the degree of cell aggregation measured as described in materials and methods (N = 4, mean ± SD; *P < 0.05, and ***P < 0.005). (**C**) The surface expression of N-cadherin on HT1080 stable cells as analyzed by flow cytometry analysis. MFI: mean fluorescence intensity. (**D**) Western blotting analysis of the total N-cadherin. The relative intensity of N-cadherin band on the western blot was determined by densitometer. (The exact p-value numbers for each comparison are listed in the supplemental materials).

Cell survival in apoptotic conditions depends on the expression and activation of multiple cellular proteins and signaling pathways^34,35^. One of the factors that affects cell survival is N-cadherin and the signaling induced by its homophilic interaction^36^. Our previous publication has shown that CD97/WT-expressing HT1080 cells up-regulated the expression of N-cadherin in serum-free conditions, which in turn induced homotypic cell-cell aggregation^21^. To examine whether the RGD motif plays a role in CD97-upregulated N-cadherin expression and its relation to cell apoptosis, we first performed the cell aggregation assay. As shown, cells expressing CD97/WT, CD97/RGE, EMR2/RGD, and EMR2/RGD-CD97/7TM aggregated more quickly and formed larger aggregates (~60% cell aggregation) than control cells and EMR2/WT-expressing cells (~20% cell aggregation) (Fig. 6A,B). Consistently, the flow cytometry and western blot analyses showed a ~2-fold increase of N-cadherin expression in cells expressing CD97/WT and CD97/RGE and a ~1.6-fold increase in cells expressing EMR2/RGD and EMR2/RGD-CD97/7TM in comparison to the control cells and EMR2/WT-expressing cells (Fig. 6C,D).

**Figure 7:**
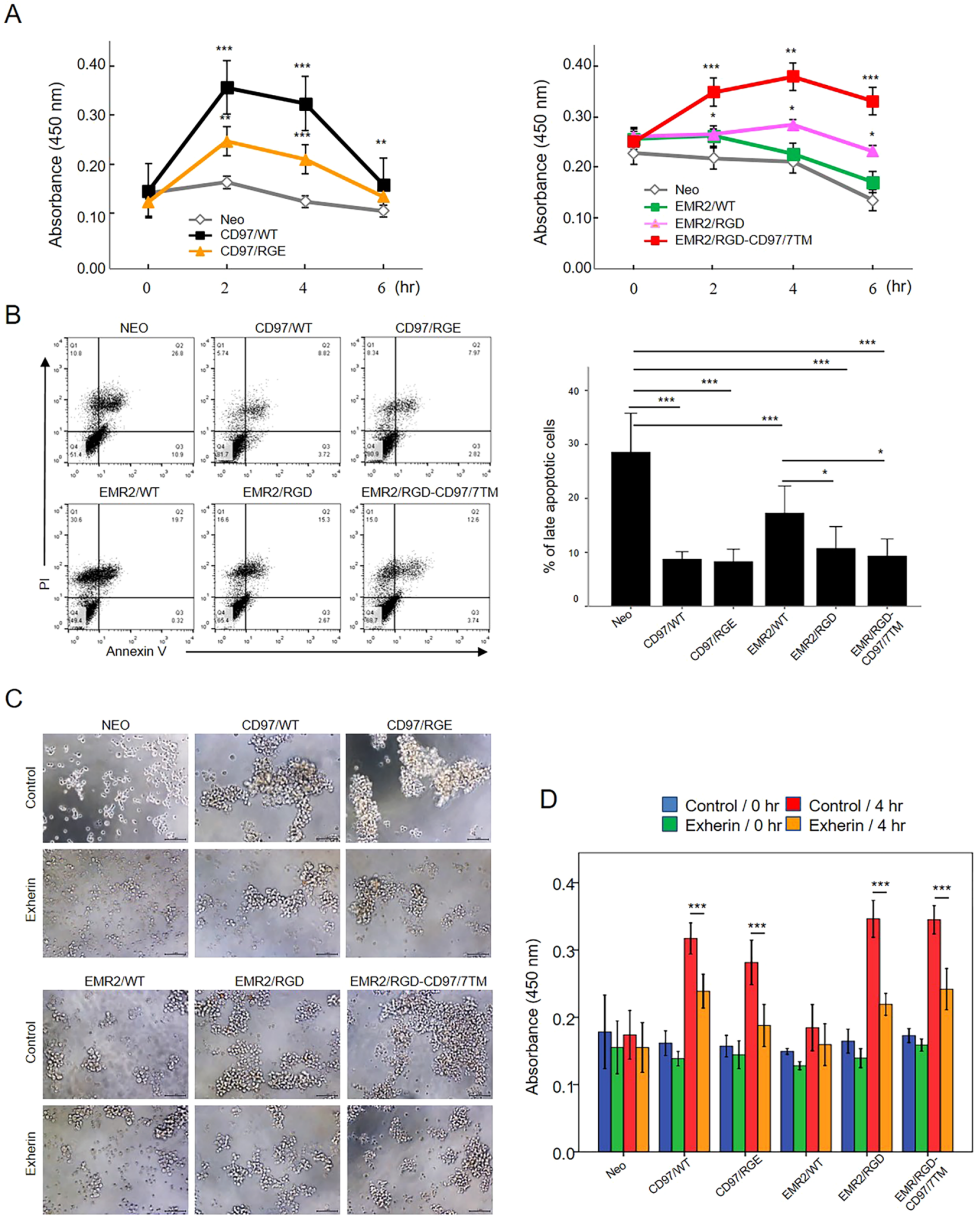
Enhanced cell aggregation promoted by up-regulated N-cadherin inhibits HT1080 cell apoptosis. (**A**) Cell viability analysis of indicated HT1080 stable cells incubated in serum-free conditions that favour N-cadherin mediated cell aggregation. Cell viability was quantified using the WST-1 cell proliferation assay kit. (N = 4, mean ± SD; *P < 0.05, **P < 0.01 and ***P < 0.005 versus HT1080-Neo control cells). (**B**) The flow cytometry analysis of cell apoptosis of indicated stable HT1080 cells. Cells were cultured in serum-free conditions that favour N-cadherin mediated cell aggregation for 4 h. Left panel: the AV/PI staining patterns determined by the FACS analysis. Right panel: a graph showing the percentage of late apoptotic (AV^+^/PI^+^) cells (N = 4, mean ± SD; ***P < 0.005). (**C**) Microscopy observation of homotypic cell aggregation of indicated HT1080 stable cells cultured in the absence or presence of Exherin (100 μg/ml) at 4 h incubation. Scale bar: 100 μm. (**D**) Cell viability analysis of HT1080 stable cells as described in (**C**). Cell viability was quantified using the WST-1 cell proliferation assay kit at 0 and 4 h incubation. (N = 3, mean ± SD; ***P < 0.005). (The exact p-value numbers for each comparison are listed in the supplemental materials).

Next, cell viability was determined in culture conditions that promoted homotypic N-cadherin-mediated cell aggregation. In accordance with the N-cadherin expression level, cells expressing CD97/WT, CD97/RGE, EMR2/RGD, and EMR2/RGD-CD97/7TM displayed a significantly higher viability than did the control cells and EMR2/WT-expressing cells (Fig. 7A). As expected, the apoptosis assay analyzed at 4 h of cell aggregation induction showed a much higher percentage of apoptotic cells in the control cells (28.55%) and EMR2/WT-expressing cells (17.25%) than cells expressing CD97/WT (8.72%), CD97/RGE (8.28%), EMR2/RGD (10.71%), and EMR2/RGD-CD97/7TM (9.3%) (Fig. 7b). Similar results were also obtained at 2 h of cell aggregation induction (Supplementary Fig. 3). In summary, cell aggregation induced by homophilic N-cadherin interaction seems to protect cell from intrinsic apoptosis.

To further confirm the anti-apoptotic role of N-cadherin, a selective N-cadherin competitor, the ADH-1 cyclic pentapeptide (Exherin), was included in the cell aggregation experiment. Interestingly, smaller cell aggregates were formed in the presence of Exherin, which resulted in significantly reduced cell viability in cells expressing CD97/WT, CD97/RGE, EMR2/RGD, and EMR2/RGD-CD97/7TM (Fig. 7C,D). By contrast, Exherin showed no significant effect on the cell growth of HT1080-Neo control cells and EMR2/WT expressing cells (Fig. 7D). Of note, the inhibitory effect of Exherin is specific but dose-dependent as the use of a control GRADSP peptide and lower concentration of Exherin did not show any obvious changes in all stable cells tested (Supplementary Fig. 4). Taken together, we conclude that the surface N-cadherin levels and the degree of cell aggregation are a positive determinant in inhibiting intrinsic apoptosis induced by serum starvation of the HT1080 stable cells.

## Discussion

The aGPCR CD97 is expressed and/or modified aberrantly in many human cancerous tissues and functionally involved in multiple tumorigenic processes including cell migration, invasion, apoptosis and angiogenesis^1,19^. For example, CD97 was recently found to promote the migration, invasion, and metastasis of hepatocellular carcinoma cells via the interaction with the G protein-coupled receptor kinase 6 (GRK6)^37^. Our previous data have indicated an absolute requirement of the GPS proteolysis, the complete 7TM region as well as the proper NTF-CTF interaction for the CD97-modulated tumorigenic functions^21–23^.

In the current study we highlight the importance of the RGD motif located at the CD97-NTF by multiple approaches including site-directed mutants, chimeric receptor, and function-blocking peptides. Interestingly, the RGD sequence on CD97 is not found in all species. Mouse and rat CD97 do not have the RGD motif ^38^. By contrast, human, gorilla, and chimpanzee, but not monkey and orangutan CD97 molecules contain the RGD motif. These findings strongly suggest a unique functional role of the RGD tripeptide for the CD97 receptor in selective primate species.

The functional role of the RGD motif of CD97 was first revealed by Kelly and colleagues who showed that CD97 promoted *in vivo* angiogenesis by interacting with the α5β1 and αvβ3 integrins on HUVECs via this unique tri-peptide sequence^15^. Our present report provides additional evidence for the significance of the RGD motif in modulating tumor cell adhesion and apoptosis mediated by CD97.

We first show that the RGD motif is essential for the CD97-promoted cell adhesion to plastic and various ECM proteins. Most importantly, a gain-of-function phenotype is observed in cells expressing EMR2/RGD and EMR2/RGD-CD97/7TM, but not those expressing EMR2/WT. The facts that the enhanced cell adhesion is specifically attenuated by the integrin-blocking RGD-based peptide and is correlated with the up-regulated levels of the α2β1 and αvβ5 integrins are intriguing because these two integrins are known to interact with many different ECM proteins via the RGD sequence^27–32^. It will be of interest in the future to determine whether CD97 can interact with the α2β1 and αvβ5 integrins directly, similar to its interaction with the α5β1 and αvβ3 integrins, via the RGD motif.

As both the RGD peptide and the αvβ5 integrin are known to regulate cell apoptosis^33,39,40^, it is hence not surprising that we found CD97/WT- and EMR2/RGD-expressing cells are protected from TNF/CHX-induced extrinsic apoptosis. Nevertheless, our results seem to suggest the underlying anti-apoptotic mechanism is likely due to the RGD motif-dependent up-regulation of αvβ5 integrin and the resulting enhanced cell adhesion. As the integrin-mediated cell adhesion usually induced an outside-in signalling, how these signalling events attenuate extrinsic apoptosis merits further investigation in later studies.

In contrast to its involvement in extrinsic apoptosis, the anti-apoptotic role of the RGD motif in cells under serum deprivation-induced intrinsic apoptosis appears much less evident. This conclusion is based on the finding that both CD97/WT- and CD97/RGE-expressing cells displayed similar growth advantage in intrinsic apoptotic conditions. In fact, our analyses suggest the levels of surface N-cadherin and cell aggregation induced by N-cadherin are critical in attenuating serum starvation-induced intrinsic apoptosis. In this scenario, the RGD motif on CD97 does not seem essential because a similar enhanced N-cadherin level and degree of cell-cell aggregation were detected in both the CD97/WT- and CD97/RGE-expressing cells. On the other hand, both EMR2/RGD and EMR2/RGD-CD97/7TM stable cells expressed a higher level of N-cadherin and became more readily aggregated when compared to EMR2/WT cells. As expected, these two groups of EMR2/RGD cells also showed a better growth advantage than the control cells and EMR2/WT cells. Hence, it is concluded that the RGD motif on CD97 is not involved directly in the anti-apoptotic effect of cells in intrinsic apoptosis. Interestingly, it was shown by others recently that tumor CD97 interacted with and activated platelets, leading to vascular permeability and tumor invasion via an RGD-independent reaction^41^. In contrast, the RGD sequence has a critical influence on the anti-apoptotic function of EMR2 by promoting N-cadherin expression and leading to homotypic cell aggregation. The questions remain whether the same or different signaling events mediated by CD97/WT and EMR2/RGD induce N-cadherin expression?

In conclusion, our results herein unravel the specific role of the RGD motif on CD97-mediated tumorigenic functions, namely tumor cell adhesion and apoptosis. We show that CD97 enhances tumor cell adhesion via a RGD-dependent mechanism, but promotes an anti-apoptotic function via a RGD-dependent and RGD-independent process in extrinsic and intrinsic apoptotic conditions, respectively. These findings provide an in-depth understanding of CD97-modulated tumor cell adhesion and apoptosis, and might be helpful in the future development of specific reagents targeting CD97-regulated tumor progression.

## Materials and Methods

### Reagents and antibodies

Cycloheximide, the GRGDSP and GRADSP peptides, and the ECM proteins fibronectin from bovine plasma (F1141) and laminin from Engelbreth-Holm-Swarm murine sarcoma basement membrane (L2020) were from Sigma-Aldrich (St. Louis, MO, USA). Collagen I from rat tail (354236) and collagen IV from Engelbreth-Holm-Swarm lathrytic mouse tumor (354233) were from Corning. All integrin antibodies, including those for the active β1 subunit (MAB2079Z, clone HUTS-4), α2β1 (MAB1998Z, clone BHA2.1), αvβ5 (MAB1961Z, clone P1F6), and anti-LIBS2 epitope (beta3 subunit) (MABT27, clone ab62), were from Millipore (Bedford, MA, USA). TNF-α was purchased from PeproTech (NJ, USA). Exherin (ADH-1)(HY-13541) was obtained from MedChemExpress (NJ, USA). CLB/CD97-1 (mouse IgG2a) and 2A1 (mouse IgG1) monoclonal antibodies (mAbs) were gifts from Dr. Jörg Hamann (University of Amsterdam, Netherlands). The anti N-cadherin (clone 32) mAb was from BD Biosciences (San Jose, CA, USA). Monoclonal antibodies used for the western blotting analysis of apoptosis including anti-MCl-1 (clone D35A5), anti-BAD (clone D24A9), anti-BAK (clone D2D3), anti-PUMA (clone D30C10), anti-caspase-3 (clone 3G2), anti-cleaved caspase-3 (clone D175), anti-caspase-9 (human specific, catalogue no. 9502), and anti-PARP-1 (catalogue no. 9542) were from Cell Signaling Technology (MA, USA).

### Vector construction

All expression vectors were constructed using the pFB-Neo vector (Stratagene, La Jolla, CA). The constructs expressing the CD97- and EMR2-WT proteins were described previously^23^. The CD97/RGE and EMR2/RGD expression constructs were made by site-directed mutagenesis of the corresponding WT constructs using specific primers: CD97/A-R (5′-GTTCTTTTCCCCCCGCTCCTGGATCA-3′) and CD97/A-F (5′-CGGGGGGAAAAGAACGTCACTATGGG-3′) for CD97/RGE and EMR2-RGD-R (5′-GTCACCCCTTTTCTGTGCCTGATTCC-3′) and EMR2-RGD-F (5′-AGGCACAGAAAAGGGGTGACCCAGGCCCTTC-3′) for EMR2/RGD. The EMR2/RGD-CD97/7TM chimeric protein expression construct was generated by digesting and ligating the EMR2/RGD pFB-Neo vector and the 7TM region of CD97 via the *Bam*HI and *Not*I sites. The fidelity of cDNA fragments of all constructs were verified by DNA sequencing.

### Retroviral infection and selection of stable HT1080 cells

HT1080 cells stably expressing CD97(1–5)/WT and EMR2(1–5)/WT were described previously^21,23^ and the stable HT1080 cells over-expressing CD97(1–5)/RGE, EMR2(1–5)/RGD and EMR2/RGD-CD97/7TM were generated using the same protocol. Briefly, HEK-293T cells were transfected with 3 µg each of pVpack-GP, pVpack-VSV-G (Stratagene, La Jolla, CA) and the designated pFB-Neo expression construct. Virus-containing supernatant was collected two days post-transfection for the transduction of HT1080 cells. Drug-resistant stable cells were selected and maintained in Minimum Essential Medium (MEM) containing 0.5 mg/ml G418.

### Western blotting and flow cytometry analyses

Western blotting analysis was carried out essentially as described^21^. In brief, protein lysates were separated in 10% SDS-PAGE gels, blotted, and probed with 5 µg/ml specific primary antibody. The membrane was then incubated with horseradish peroxidase (HRP)-conjugated goat anti-mouse secondary antibody (1:2500) (Sigma). The protein bands were visualized and captured using the BioSpectrum 610 Imaging System (UVP, Upland, CA, USA). For flow cytometry analysis, cells were fixed in 2% paraformaldehyde in PBS for 20 min at 4 °C and sequentially blocked with blocking buffer (PBS containing 5% goat normal serum and 1% BSA) for 1 h at 4 °C. Cells were stained with the appropriate first antibody (5 µg/ml) and second antibody (1:200) in blocking buffer for 1 hour at 4 °C. Cells were analyzed on a FACS Calibur (BD Biosciences).

### Cell viability, adhesion, and aggregation assays

Cell viability was determined either using the WST-1 cell proliferation assay kit (Sigma-Aldrich) or by counting the number of live cells with a haemocytometer. Cell adhesion and cell aggregation assays were conducted as described previously^21,23^. In short, cells were incubated in normal culture plastics without coating or pre-coated with specific ECM proteins, such as fibronectin (0.2 µg/ml), laminin (0.4 µg/ml), collagen I (0.04 µg/ml) and collagen IV (0.25 µg/ml). HT1080 stable cells were serum-starved for 20 h and then seeded in 96-well plates (10^5^ cells/100 µl/well) for 1 h at 37 °C. Non-adherent cells were washed away carefully with HBSS and the attached cells were fixed with 2% glutaraldehyde in 0.1 M sodium cacodylate trihydrate (pH 7.2) buffer for 20 min. Following extensive washes, attached cells were stained with 1% methylene blue in 0.01 M sodium tetraborate buffer for 30 min. Excess dye was washed off with water, and cells were lysed with 75% ethanol to be measured at 595 nm (TECAN, M€annedorf, Switzerland)^23^. When necessary, cells were incubated in the presence of the GRGDSP or GRADSP peptide (50 µg/ml).

For the cell aggregation assay, serum-starved stable HT1080 cells (5 × 10^5^ cells/mL) were suspended in serum-free MEM containing 5% BSA and incubated in 96-well plates (100 µl/well) for 1 h at 37 °C. Subsequently, cells were fixed with 1% formaldehyde in PBS and counted with a hemocytometer. The equation to determine the percentage of cell aggregation was (1 − (N1/N0))×100% where N1 is the number of single cells after incubation and N0 is the number of single cells at the beginning of the experiment.

### Apoptosis assay

The intrinsic and extrinsic cellular apoptotic pathways were induced by culturing cells in serum-free medium for 48 h and in the presence of cycloheximide (10 μg/ml) and TNF-α (5 ng/ml) for 6 h, respectively. Cell apoptosis was determined using the TACS® Annexin V-FITC kit (Trevigen, MD, USA) as described previously^22^.

### RT-PCR and Quantitative real-time PCR analysis

Total RNA was isolated from cells underwent 20 h starvation treatment using TRI reagents [Sigma-Aldrich]. Isolated RNA was treated with RNAse-free DNAse [Promega]. The DNA-free total RNAs were reverse transcribed using oligo(dT)_18_ and MMLV reverse transcriptase [Invitrogen] according to manufacturer’s instructions. The quantitative real-time PCR analysis was performed using iQ SYBR Green Supermix [BioRad]. Complementary DNA is amplified by gene-specific primers Human αv integrin, 5′-GGCGATGGCGTAGATGACTT-3′, 5′-GGCGCTCCGATGAACACAT-3′, and human β5 integrin, 5′-TCAGATGGACTATCCGTCCCTT-3′, 5′-TTGTCCCAGGTATCAGGGCT-3′^42^. The results were analyzed by LightCycler®96 software [Roche] and normalized to internal control GAPDH.

### Small interfering RNA transfection

Small interfering RNAs (siRNAs) were transfected using the Lipofectamine RNAiMAX Transfection Reagent (ThermoFisher Scientific) according to manufacturer’s instructions. The siRNA primer to knockdown integrin β5 subunit is 5′-GCUCGCAGGUCUCAACAUA-dTdT-3′^43^.

### Statistical analysis

Results are shown as mean ± standard deviation (SD), and comparisons were made using Student’s t-test or ANOVA with post hoc LSD. A difference with a probability (P) of < 0.05 was considered significant.

## Supplementary information

Supplementary Figures, uncropped gel, and the exact P-value numbers
