# Supplementary material for: The RGD motif is involved in CD97/ADGRE5-promoted cell adhesion and viability of HT1080 cells
Source: Sci Rep. 2019 Feb 6;9:1517. doi: 10.1038/s41598-018-38045-w (PMC6365523; doi:10.1038/s41598-018-38045-w)
Supplement: Supplementary file 1 — Supplementary Figures, uncropped gel, and the exact P-value numbers [file 41598_2018_38045_MOESM1_ESM.pdf]

Supplementary Figures

The RGD motif is involved in CD97/ADGRE5-promoted cell adhesion and viability of HT1080 cells

Wen-Ye Tjong[1], Hsi-Hsien Lin[1,2,3,4,*]

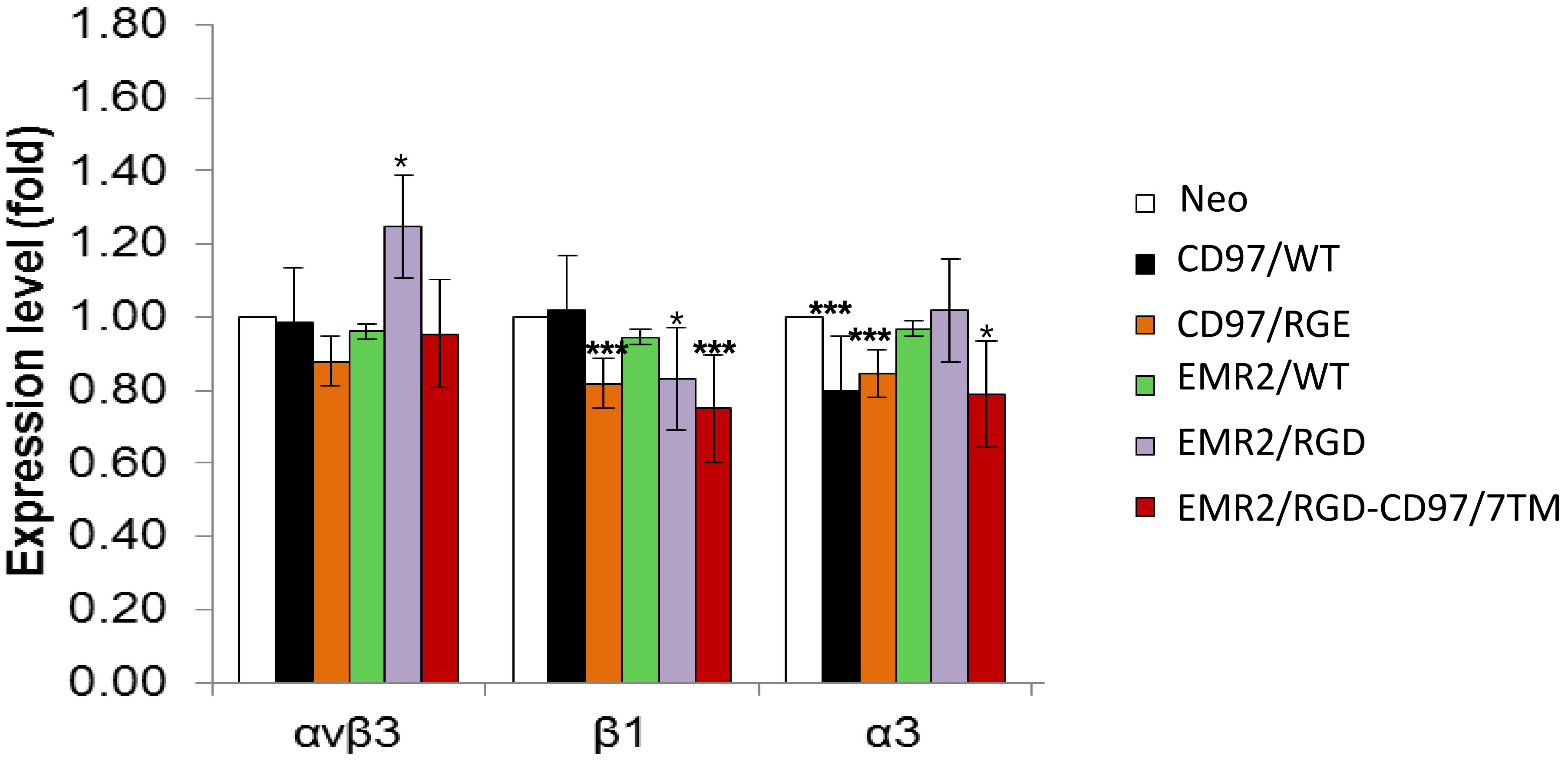

Suppplementary Fig 1. Flow cytometry analysis of the expression of selective integrin molecules on different HT1080 stable cells as indicated. N=3, mean±SD. *P < 0.05, **P < 0.01, and ***P < 0.005 versus the control HT1080-Neo cells.

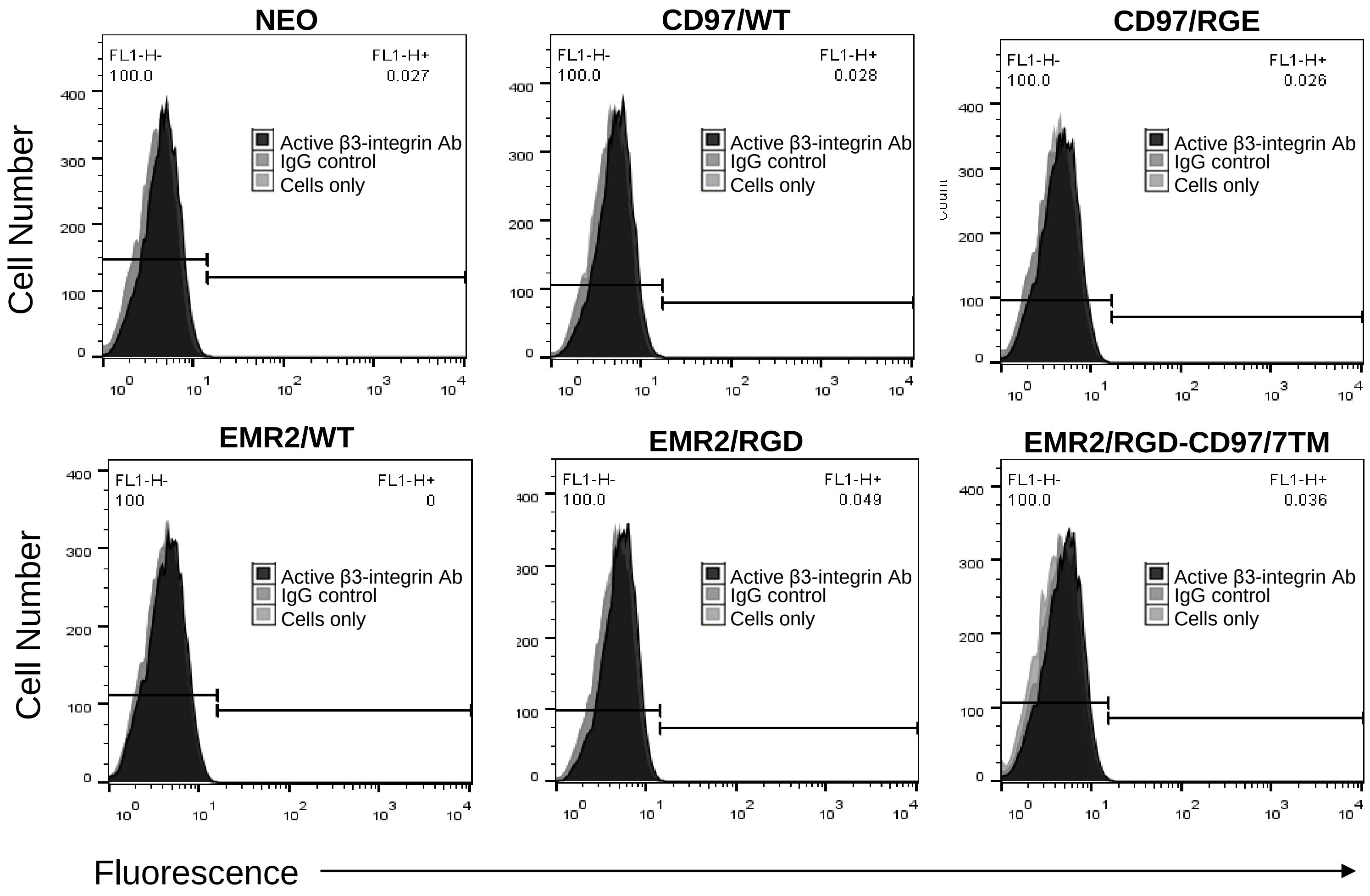

Supplementary Fig 2. The levels of active β3 integrin were examined by flow cytometry analysis after stable cells were starved overnight. The monoclonal antibody against the active β3-integrin LIBS2 epitope (MABT27) was used for the staining. The figure is the representative of four independent experiments.

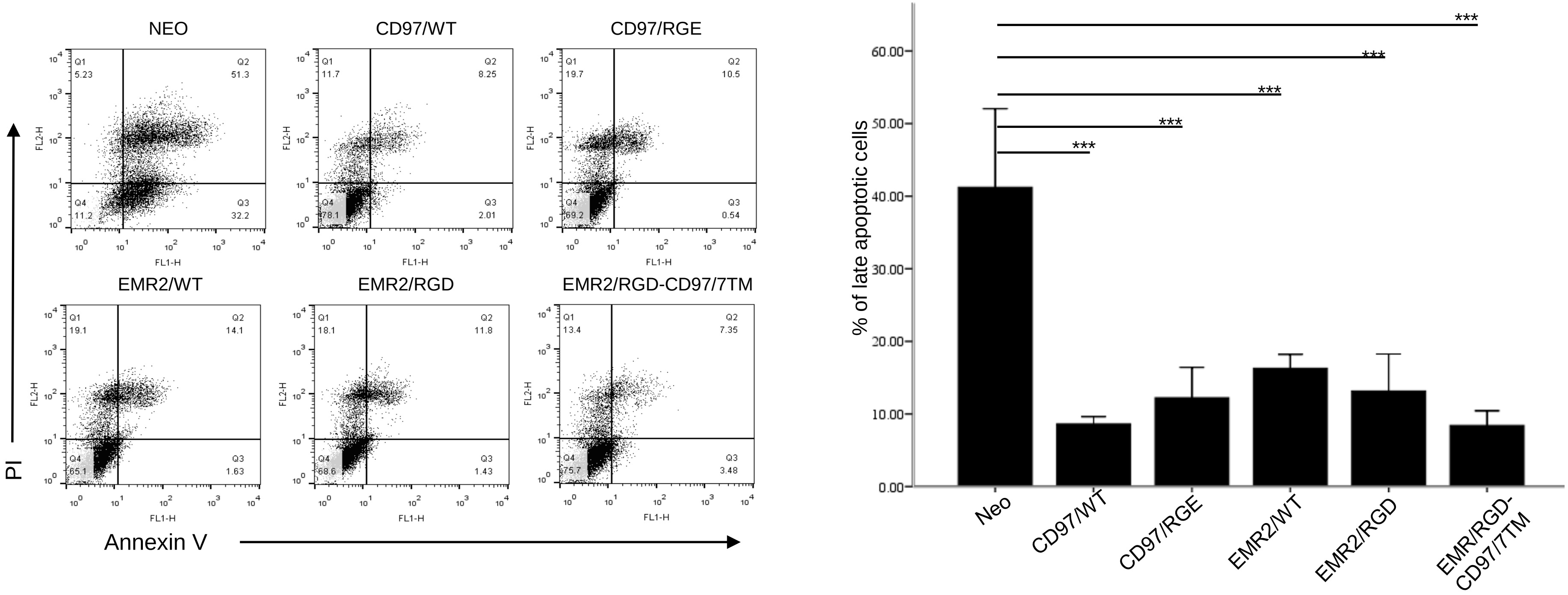

Suppplementary Fig 3. The flow cytometry analysis of cell apoptosis of indicated stable HT1080 cells. Cells were cultured in serum-free conditions that favour N-cadherin mediated cell aggregation for 2 h (left panel). The graph indicates the percentage of AV+/PI+ cells (right panel) (N = 4, mean±SD; *P < 0.05).

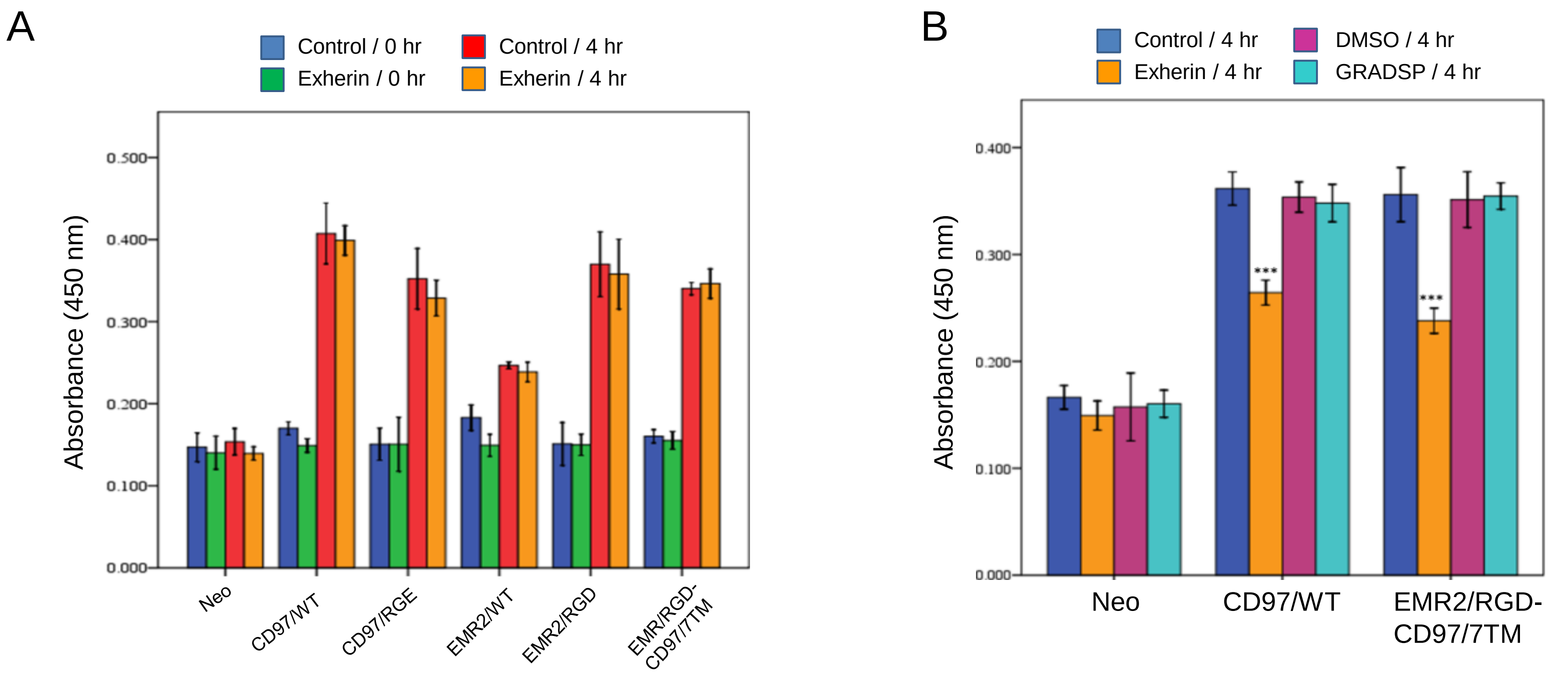

Supplementary Fig 4. N-cadherin-promoted cell aggregation inhibits HT1080 cell apoptosis. Serum-starved cells were incubated for 4 h at 37 ºC in serum-free medium containing 5% BSA in the presence of (a) Exherin (50 µg/ml), and (b) Exherin (100 µg/ml) or GRADSP peptide (100 µg/ml) or DMSO. WST-1 cell proliferation assay was used to quantify cell viability. ***P < 0.005 versus control without Exherin after 4 h incubation. Lane 1: HT1080-Neo cells; lane 2: HT1080-CD97/WT cells; lane 3: HT1080-CD97/RGE cells; lane 4: HT1080-EMR2/WT cells; lane 5: HT1080-EMR2/RGD cells; lane 6: HT1080-EMR2/RGD-CD97/7TM cells.

Figure 1

C

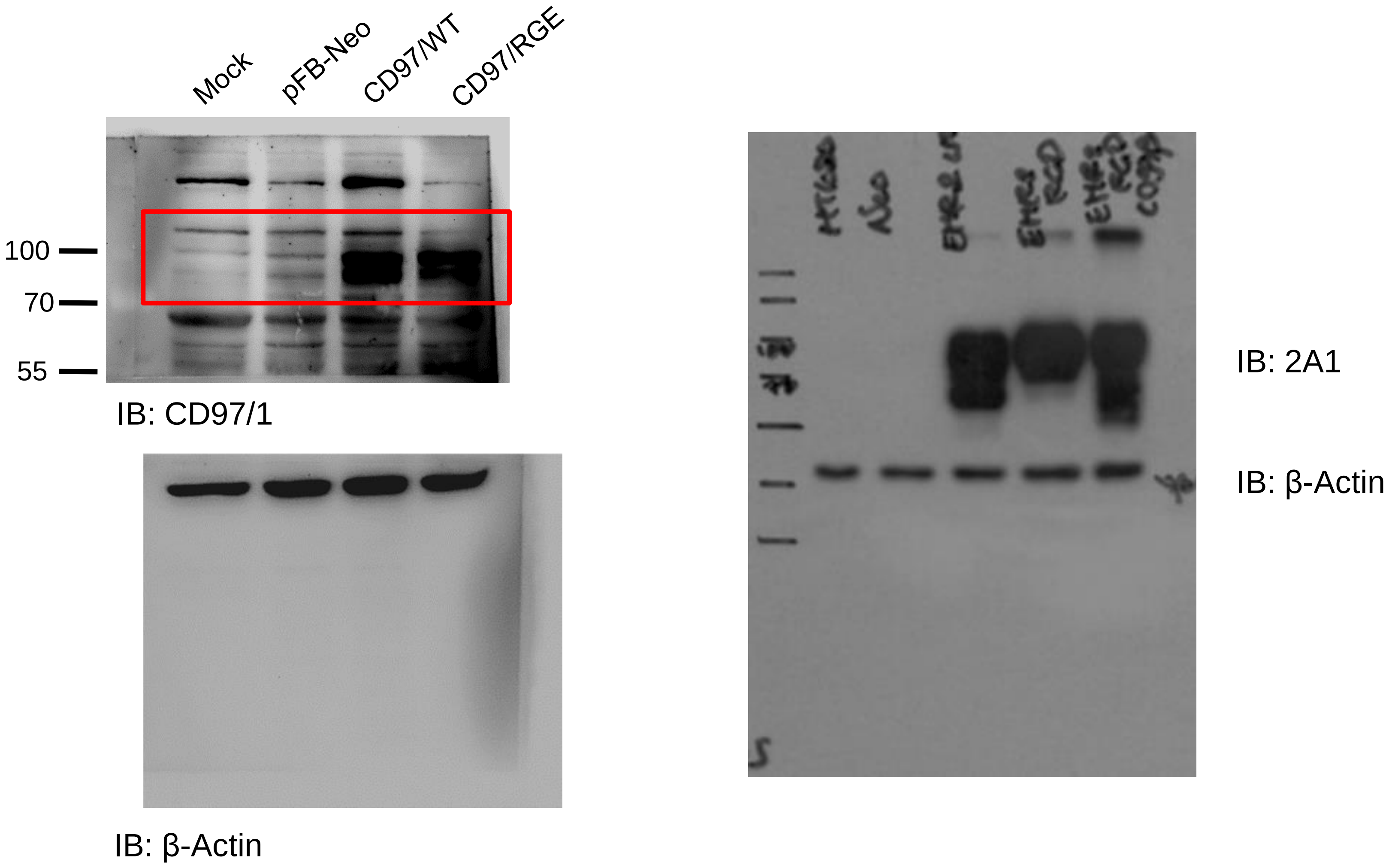
Mock
pFB-Neo
CD97/WT
CD97/RGE
100
70
55
IB: CD97/1
IB: β-Actin
IB: 2A1
IB: β-Actin

Figure 2

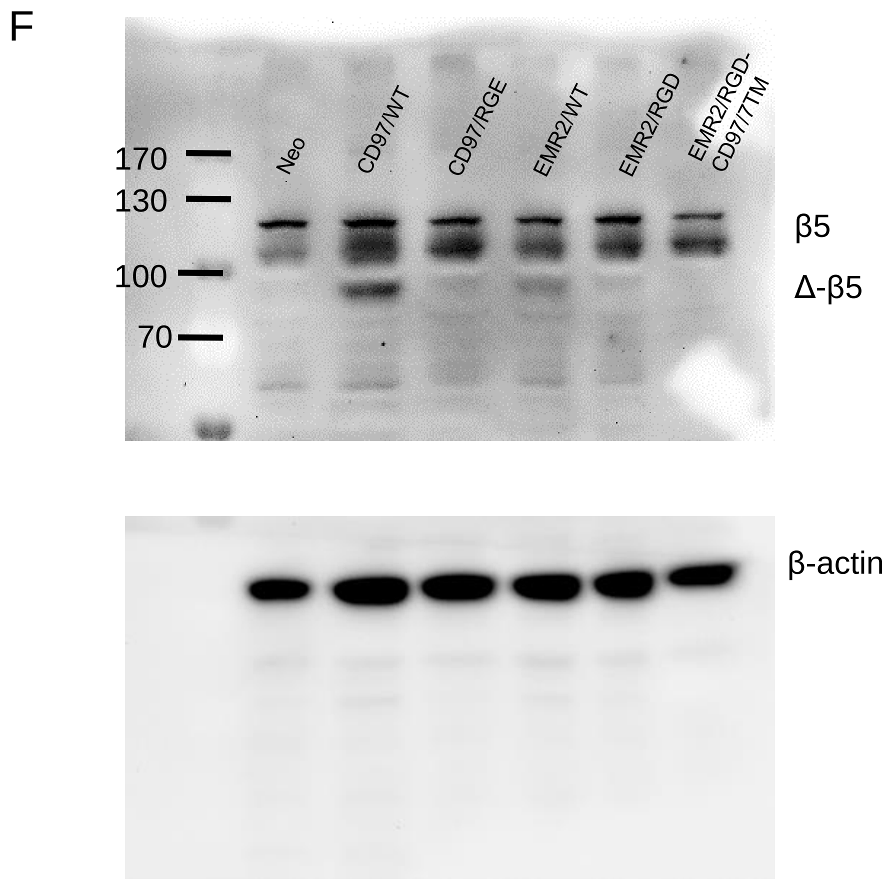
F
170
130
100
70
Neo
CD97/WT
CD97/RGE
EMR2/WT
EMR2/RGD
EMR2/RGD-
CD97/7TM
β5
Δ-β5
β-actin

The exact P-value numbers of each comparison are listed below

Figure 2B

Plastic:
Neo vs CD97/WT ($P = 1.08x10^{-8}$)
Neo vs CD97/RGE ($P = 0.0178$)
Neo vs EMR2/RGD ($P = 6.65x10^{-4}$)
Neo vs EMR2/RGD-CD97/7TM ($P = 5x10^{-6}$)
CD97/WT vs CD97/RGE ($P = 3.75x10^{-7}$)
EMR2/WT vs EMR2/RGD ($P = 4.423x10^{-3}$)
EMR2/WT vs EMR2/RGD-CD97/7TM ($P = 2.3x10^{-5}$)
EMR2/RGD vs EMR2/RGD-CD97/7TM ($P = 0.0122$)

Collagen I:
Neo vs CD97/WT ($P = 2.3x10^{-4}$)
Neo vs EMR2/RGD-CD97/7TM ($P = 8.1x10^{-5}$)
CD97/WT vs CD97/RGE ($P = 6.69x10^{-4}$)
EMR2/WT vs EMR2/RGD-CD97/7TM ($P = 3.4x10^{-5}$)
EMR2/RGD vs EMR2/RGD-CD97/7TM ($P = 4.14x10^{-3}$)

Fibronectin:
Neo vs CD97/WT ($P = 2.5x10^{-5}$)
Neo vs EMR2/RGD ($P = 0.05$)
Neo vs EMR2/RGD-CD97/7TM ($P = 1.74x10^{-3}$)
CD97/WT vs CD97/RGE ($P = 7.26x10^{-4}$)
EMR2/WT vs EMR2/RGD ($P = 0.032$)
EMR2/WT vs EMR2/RGD-CD97/7TM ($P = 1.12x10^{-3}$)

Laminin:
Neo vs CD97/WT ($P = 4x10^{-5}$)
Neo vs EMR2/RGD ($P = 9.22x10^{-3}$)
Neo vs EMR2/RGD-CD97/7TM ($P = 1.95x10^{-4}$)
CD97/WT vs CD97/RGE ($P = 2.45x10^{-4}$)
EMR2/WT vs EMR2/RGD ($P = 0.013$)
EMR2/WT vs EMR2/RGD-CD97/7TM ($P = 2.57x10^{-4}$)
EMR2/RGD vs EMR2/RGD-CD97/7TM ($P = 0.049$)

Collagen IV:
Neo vs CD97/WT ($P = 1.75x10^{-4}$)
Neo vs EMR2/RGD ($P = 4.42x10^{-3}$)
Neo vs EMR2/RGD-CD97/7TM ($P = 5x10^{-6}$)
CD97/WT vs CD97/RGE ($P = 5x10^{-6}$)
EMR2/WT vs EMR2/RGD ($P = 0.036$)
EMR2/WT vs EMR2/RGD-CD97/7TM ($P = 1.9x10^{-5}$)
EMR2/RGD vs EMR2/RGD-CD97/7TM ($P = 2.17x10^{-3}$)

The exact P-value numbers of each comparison are listed below

Figure 2C

Neo	GRGDSP vs GRADSP ($P = 0.04$)
CD97/WT	GRGDSP vs GRADSP ($P = 2.14X10^{-4}$)
EMR2/RGD	GRGDSP vs GRADSP ($P = 0.042$)
EMR2/RGD-CD97/7TM	GRGDSP vs GRADSP ($P = 6.22X10^{-5}$)

Figure 2D

Neo vs CD97/WT ($P = 4.95x10^{-3}$)
Neo vs EMR2/RGD ($P = 7.08x10^{-3}$)
Neo vs EMR2/RGD-CD97/7TM ($P = 0.034$)
CD97/WT vs CD97/RGE ($P = 0.015$)
EMR2/WT vs EMR2/RGD ($P = 7.8x10^{-3}$)
EMR2/WT vs EMR2/RGD-CD97/7TM ($P = 0.042$)

Figure 2E

Neo vs CD97/WT ($P = 1.65x10^{-3}$)
Neo vs EMR2/RGD ($P = 0.016$)
Neo vs EMR2/RGD-CD97/7TM ($P = 0.02$)
CD97/WT vs CD97/RGE ($P = 2.5x10^{-3}$)

The exact P-value numbers of each comparison are listed below

Figure 2G

Neo vs CD97/WT ($P = 5x10^{-6}$)
Neo vs CD97/RGE ($P = 5.22x10^{-3}$)
Neo vs EMR2/RGD-CD97/7TM ($P = 7.1x10^{-5}$)
CD97/WT vs CD97/RGE ($P = 8.96x10^{-4}$)
EMR2/WT vs EMR2/RGD-CD97/7TM ($P = 3.3x10^{-5}$)
EMR2/RGD vs EMR2/RGD-CD97/7TM ($P = 2.93x10^{-4}$)

Figure 2H

Neo vs CD97/WT ($P = 8.09x10^{-3}$)
Neo vs EMR2/WT ($P = 1.17x10^{-3}$)
Neo vs EMR2/RGD-CD97/7TM (P = 0.04)
CD97/WT vs CD97/RGE ($P = 7.9x10^{-5}$)
EMR2/WT vs EMR2/RGD ($P = 2.37x10^{-4}$)
EMR2/WT vs EMR2/RGD-CD97/7TM ($P = 5x10^{-6}$)

Figure 2J

Neo	siRNA Neg vs siRNA β5 (P = 0.013)
CD97/WT	siRNA Neg vs siRNA β5 ($P = 9.21X10^{-4}$)
EMR2/WT	siRNA Neg vs siRNA β5 (P = 0.03)
EMR2/RGD	siRNA Neg vs siRNA β5 ($P = 3.05X10^{-4}$)
EMR2/RGD-CD97/7TM	siRNA Neg vs siRNA β5 (P = 0.016)

The exact P-value numbers of each comparison are listed below

Figure 3A

4hrs:	Neo vs CD97/WT ($P = 3.1x10^{-5}$)
	Neo vs EMR2/RGD ($P = 3x10^{-3}$)
	Neo vs EMR2/RGD-CD97/7TM ($P = 3.86x10^{-3}$)
	CD97/WT vs CD97/RGE ($P = 8.36x10^{-4}$)
	EMR2/WT vs EMR2/RGD ($P = 0.01$)
	EMR2/WT vs EMR2/RGD-CD97/7TM ($P = 0.013$)
6hrs:	Neo vs CD97/WT ($P = 4.51x10^{-4}$)
	Neo vs CD97/RGE ($P = 9.41x10^{-5}$)
	Neo vs EMR2/RGD ($P = 8.8x10^{-3}$)
	Neo vs EMR2/RGD-CD97/7TM ($P = 0.14$)
	CD97/WT vs CD97/RGE ($P = 4.46x10^{-7}$)
	EMR2/WT vs EMR2/RGD ($P = 2.43x10^{-3}$)
	EMR2/WT vs EMR2/RGD-CD97/7TM ($P = 3.63x10^{-3}$)

Figure 3B

CD97/WT	GRGDSP vs GRADSP ($P = 0.014$)
EMR2/RGD	GRGDSP vs GRADSP ($P = 2.88X10^{-3}$)
EMR2/RGD-CD97/7TM	GRGDSP vs GRADSP ($P = 1.11X10^{-3}$)

The exact P-value numbers of each comparison are listed below

Figure 4A

Neo vs CD97/WT ($P = 2.83x10^{-7}$)
Neo vs CD97/RGE ($P = 2.18x10^{-4}$)
Neo vs EMR2/WT ($P = 8.82x10^{-3}$)
Neo vs EMR2/RGD ($P = 4x10^{-7}$)
Neo vs EMR2/RGD-CD97/7TM ($P = 1.02x10^{-6}$)
CD97/WT vs CD97/RGE ($P = 2.13x10^{-11}$)
EMR2/WT vs EMR2/RGD ($P = 2.86x10^{-4}$)
EMR2/WT vs EMR2/RGD-CD97/7TM ($P = 8.15x10^{-4}$)

Figure 5A

Neo vs CD97/WT ($P = 7.18x10^{-8}$)
Neo vs CD97/RGE ($P = 4.75x10^{-7}$)
Neo vs EMR2/WT ($P = 1.01x10^{-3}$)
Neo vs EMR2/RGD ($P = 4.79x10^{-7}$)
Neo vs EMR2/RGD-CD97/7TM ($P = 1.79x10^{-7}$)
EMR2/WT vs EMR2/RGD ($P = 1.53x10^{-3}$)
EMR2/WT vs EMR2/RGD-CD97/7TM ($P = 7.47x10^{-4}$)

Figure 4B

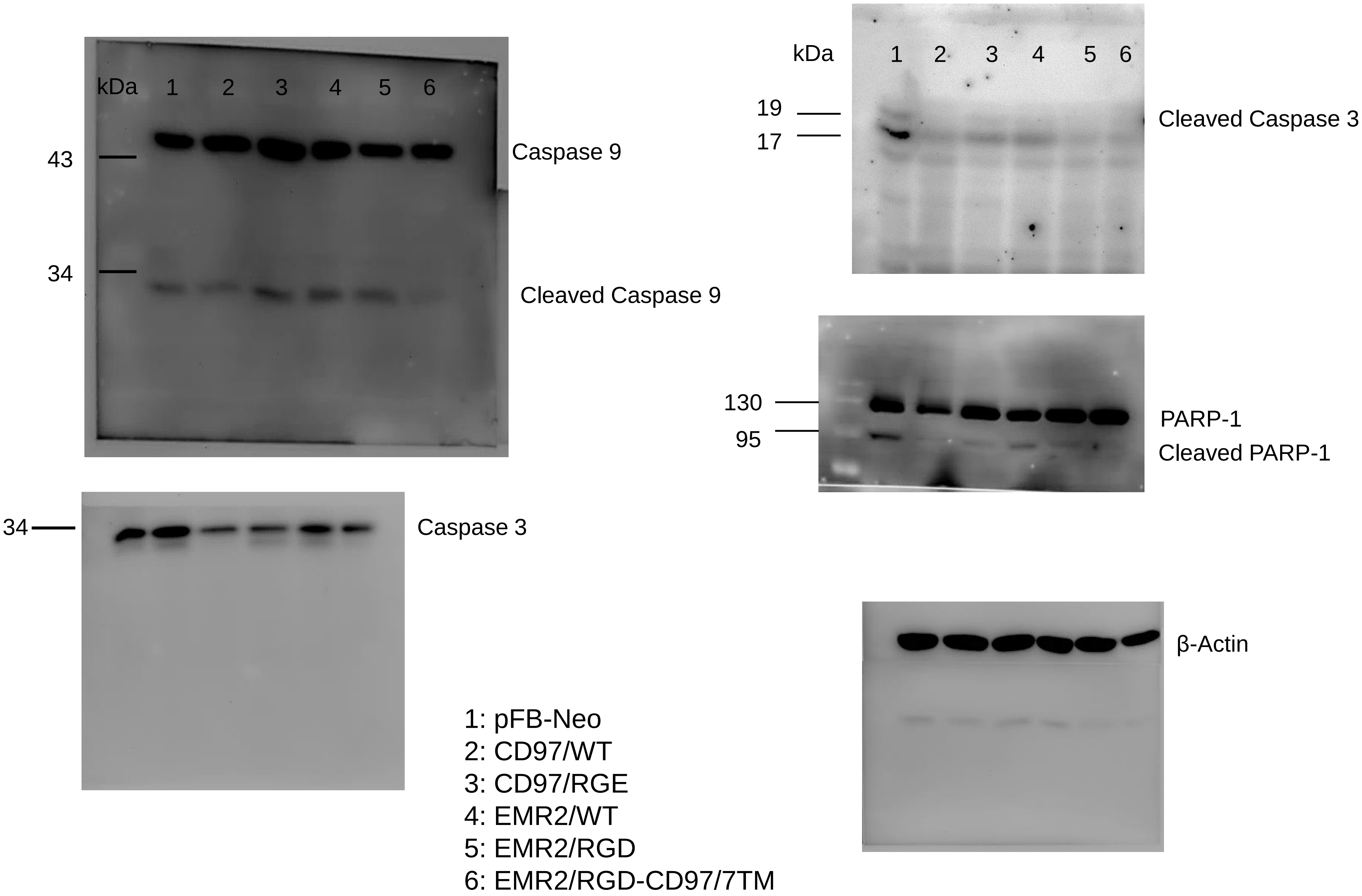
kDa 1 2 3 4 5 6
43
34
Caspase 9
Cleaved Caspase 9
34
Caspase 3
kDa 1 2 3 4 5 6
19
17
Cleaved Caspase 3
130
95
PARP-1
Cleaved PARP-1
β-Actin
1: pFB-Neo
2: CD97/WT
3: CD97/RGE
4: EMR2/WT
5: EMR2/RGD
6: EMR2/RGD-CD97/7TM

Figure 4C

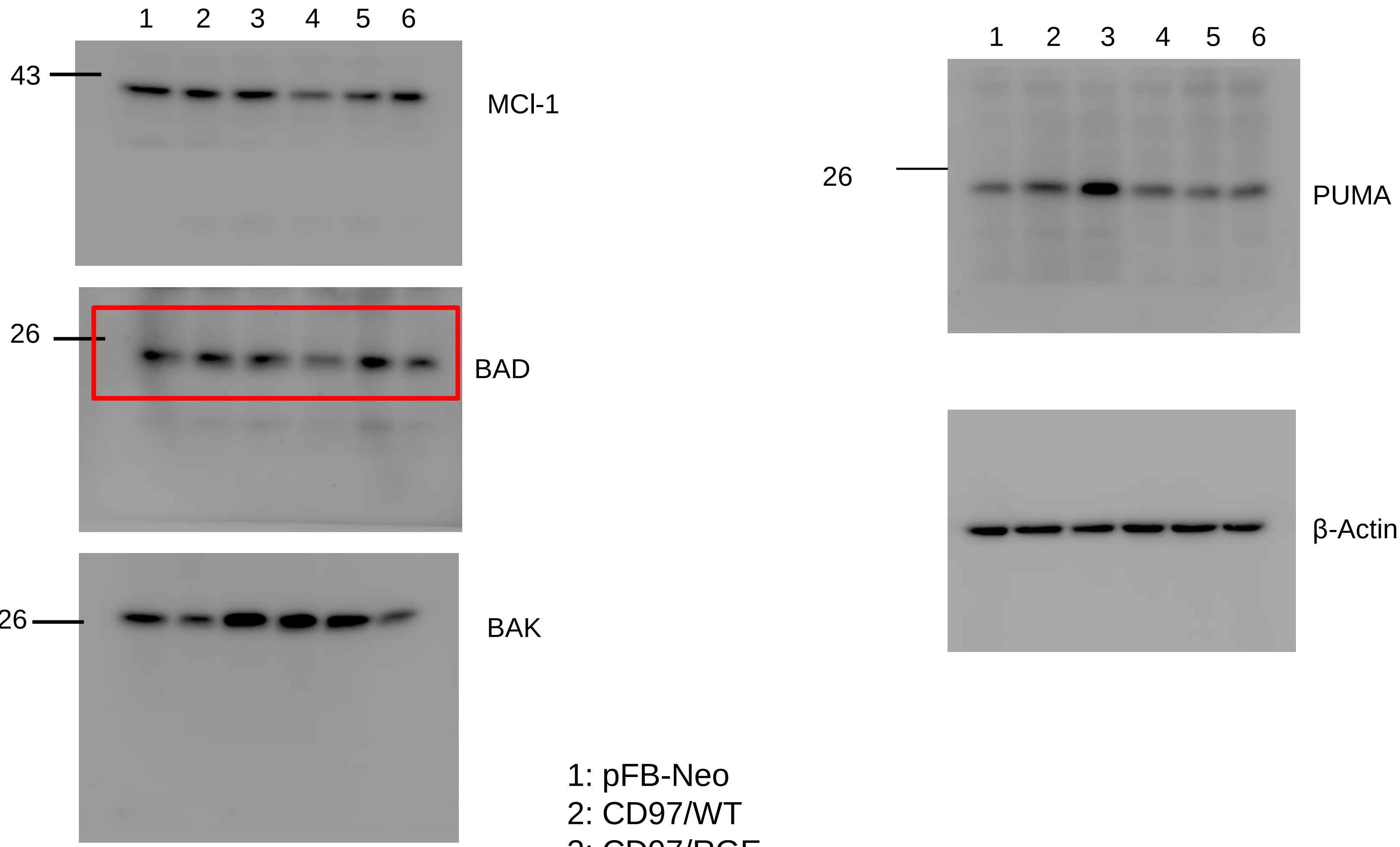

1: pFB-Neo
2: CD97/WT
3: CD97/RGE
4: EMR2/WT
5: EMR2/RGD
6: EMR2/RGD-CD97/7TM

Figure 5B

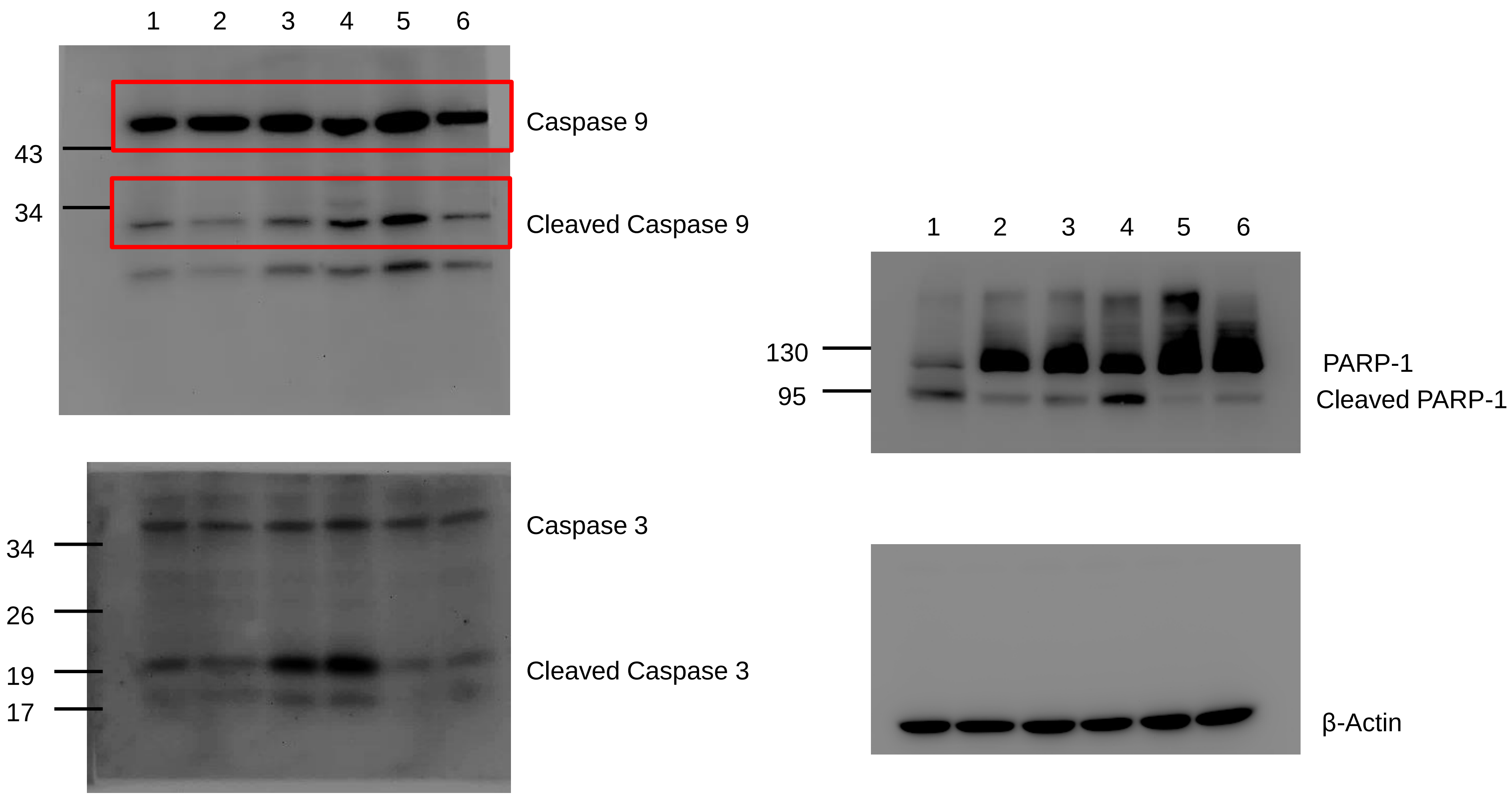

1: pFB-Neo
2: CD97/WT
3: CD97/RGE
4: EMR2/WT
5: EMR2/RGD
6: EMR2/RGD-CD97/7TM

Figure 5C

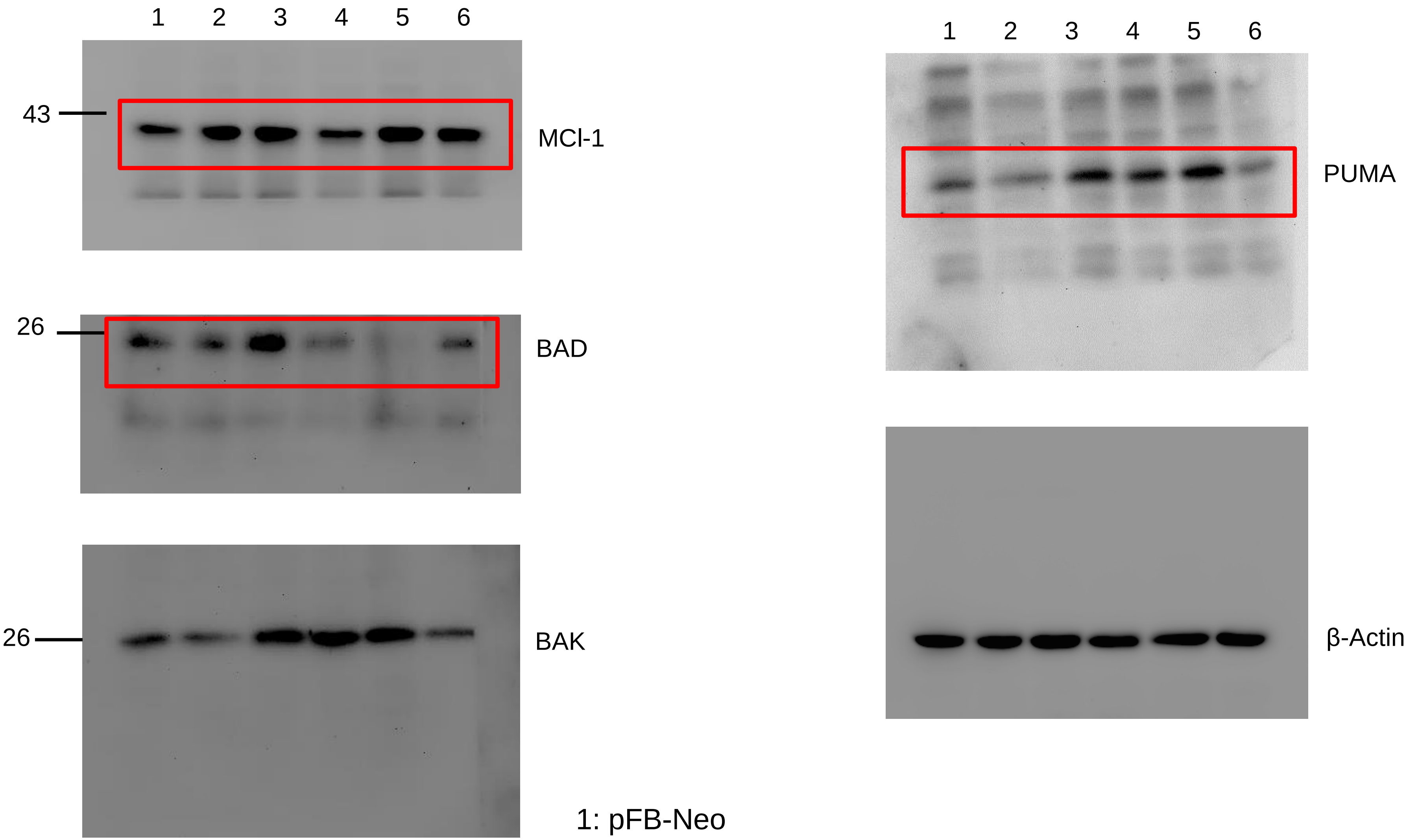

1: pFB-Neo
2: CD97/WT
3: CD97/RGE
4: EMR2/WT
5: EMR2/RGD
6: EMR2/RGD-CD97/7TM

The exact P-value numbers of each comparison are listed below

Figure 6B

Neo vs CD97/WT ($P = 1.46 \times 10^{-7}$)
Neo vs CD97/RGE ($P = 1.73 \times 10^{-6}$)
Neo vs EMR2/RGD ($P = 7.97 \times 10^{-7}$)
Neo vs EMR2/RGD-CD97/7TM ($P = 1.35 \times 10^{-7}$)
EMR2/WT vs EMR2/RGD ($P = 8.13 \times 10^{-6}$)
EMR2/WT vs EMR2/RGD-CD97/7TM ($P = 1.7 \times 10^{-6}$)

Figure 6D

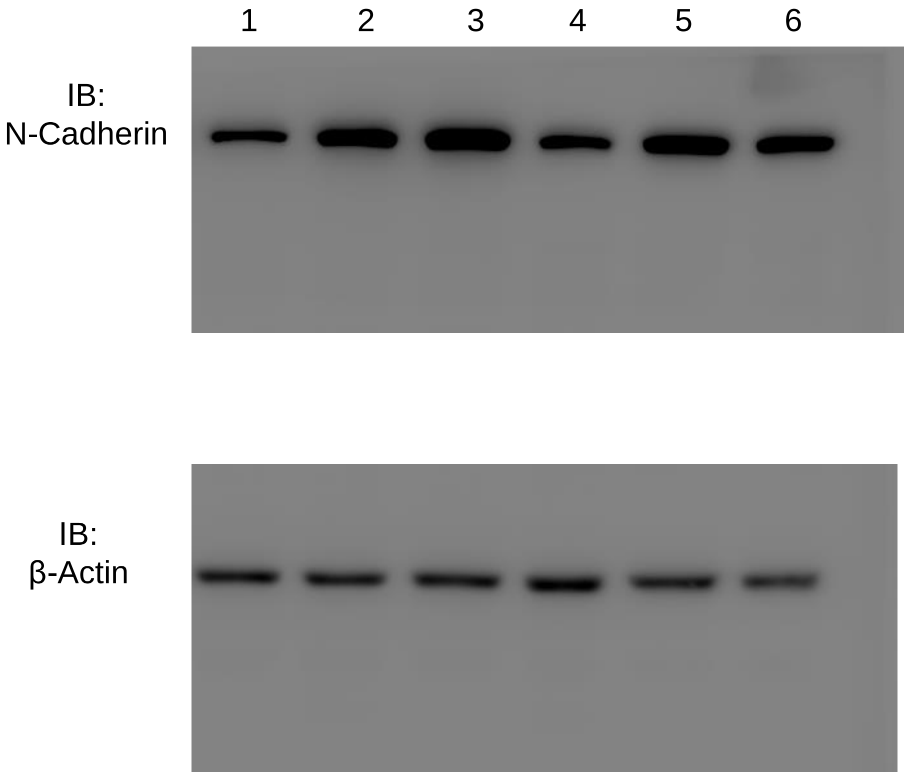

1: pFB-Neo
2: CD97/WT
3: CD97/RGE
4: EMR2/WT
5: EMR2/RGD
6: EMR2/RGD-CD97/7TM

The exact P-value numbers of each comparison are listed below

Figure 7A

2 hrs
Neo vs CD97/WT ($P = 9.46x10^{-4}$)
Neo vs CD97/RGE ($P = 6.82x10^{-3}$)
Neo vs EMR2/WT ($P = 0.027$)
Neo vs EMR2/RGD ($P = 0.02$)
Neo vs EMR2/RGD-CD97/7TM ($P = 2.8x10^{-3}$)
CD97/WT vs CD97/RGE ($P = 8.72x10^{-3}$)
EMR2/WT vs EMR2/RGD-CD97/7TM ($P = 9.8x10^{-3}$)
EMR2/RGD vs EMR2/RGD-CD97/7TM ($P = 0.011$)

4 hrs
Neo vs CD97/WT ($P = 2.5x10^{-3}$)
Neo vs CD97/RGE ($P = 2.16x10^{-4}$)
Neo vs EMR2/RGD ($P = 0.043$)
Neo vs EMR2/RGD-CD97/7TM ($P = 9.43x10^{-3}$)
CD97/WT vs CD97/RGE ($P = 0.0192$)
EMR2/WT vs EMR2/RGD-CD97/7TM ($P = 0.012$)

6 hrs
Neo vs CD97/WT ($P = 7.62x10^{-3}$)
Neo vs EMR2/RGD ($P = 0.023$)
Neo vs EMR2/RGD-CD97/7TM ($P = 3.55x10^{-7}$)
EMR2/WT vs EMR2/RGD-CD97/7TM ($P = 7.68x10^{-3}$)
EMR2/RGD vs EMR2/RGD-CD97/7TM ($P = 0.022$)

The exact P-value numbers of each comparison are listed below

Figure 7B

Neo vs CD97/WT ($P = 4.1x10^{-6}$)
Neo vs CD97/RGE ($P = 3.1x10^{-6}$)
Neo vs EMR2/WT ($P = 1.62x10^{-3}$)
Neo vs EMR2/RGD ($P = 1.5x10^{-5}$)
Neo vs EMR2/RGD-CD97/7TM ($P = 5.98x10^{-6}$)
EMR2/WT vs EMR2/RGD ($P = 0.046$)
EMR2/WT vs EMR2/RGD-CD97/7TM ($P = 0.018$)

Figure 7D

CD97/WT	Control vs Exherin β5 ($P = 3.8X10^{-3}$)
CD97/RGE	Control vs Exherin β5 ($P = 1.84X10^{-3}$)
EMR2/RGD	Control vs Exherin β5 ($P = 2.3X10^{-3}$)
EMR2/RGD-CD97/7TM	Control vs Exherin β5 ($P = 1.5X10^{-3}$)

The exact P-value numbers of each comparison are listed below

Supplementary Fig 1.

αvβ3 integrin — Neo vs EMR2/RGD (P = 0.029)

β1 integrin — Neo vs CD97/RGE (P = 1.04x10^{-5})
Neo vs EMR2/RGD (P = 0.027)
Neo vs EMR2/RGD-CD97/7TM (P = 2.53x10^{-5})

α3 integrin — Neo vs CD97/WT (P = 2.63x10^{-3})
Neo vs CD97/RGE (P = 1.56x10^{-3})
Neo vs EMR2/RGD-CD97/7TM (P = 0.046)

Supplementary Fig 3.

Neo vs CD97/WT (P = 3.03x10^{-6})
Neo vs CD97/RGE (P = 1.05x10^{-5})
Neo vs EMR2/WT (P = 4.83x10^{-5})
Neo vs EMR2/RGD (P = 1.47x10^{-5})
Neo vs EMR2/RGD-CD97/7TM (P = 1.36x10^{-6})

Supplementary Fig 4B.

CD97/WT — Control vs Exherin β5 (P = 9.52X10^{-4})
EMR2/RGD-CD97/7TM — Control vs Exherin β5 (P = 1.85X10^{-3})
